# *Neisseria* genes required for persistence identified via *in vivo* screening of a transposon mutant library

**DOI:** 10.1371/journal.ppat.1010497

**Published:** 2022-05-17

**Authors:** Katherine A. Rhodes, Man Cheong Ma, María A. Rendón, Magdalene So

**Affiliations:** 1 Immunobiology Department, University of Arizona, Tucson, Arizona, United States of America; 2 BIO5 Institute, University of Arizona, Tucson, Arizona, United States of America; INSERM, FRANCE

## Abstract

The mechanisms used by human adapted commensal *Neisseria* to shape and maintain a niche in their host are poorly defined. These organisms are common members of the mucosal microbiota and share many putative host interaction factors with *Neisseria meningitidis* and *Neisseria gonorrhoeae*. Evaluating the role of these shared factors during host carriage may provide insight into bacterial mechanisms driving both commensalism and asymptomatic infection across the genus. We identified host interaction factors required for niche development and maintenance through *in vivo* screening of a transposon mutant library of *Neisseria musculi*, a commensal of wild-caught mice which persistently and asymptomatically colonizes the oral cavity and gut of CAST/EiJ and A/J mice. Approximately 500 candidate genes involved in long-term host interaction were identified. These included homologs of putative *N*. *meningitidis* and *N*. *gonorrhoeae* virulence factors which have been shown to modulate host interactions *in vitro*. Importantly, many candidate genes have no assigned function, illustrating how much remains to be learned about *Neisseria* persistence. Many genes of unknown function are conserved in human adapted *Neisseria* species; they are likely to provide a gateway for understanding the mechanisms allowing pathogenic and commensal *Neisseria* to establish and maintain a niche in their natural hosts. Validation of a subset of candidate genes confirmed a role for a polysaccharide capsule in *N*. *musculi* persistence but not colonization. Our findings highlight the potential utility of the *Neisseria musculi*-mouse model as a tool for studying the pathogenic *Neisseria;* our work represents a first step towards the identification of novel host interaction factors conserved across the genus.

## Author summary

The *Neisseria* genus contains many genetically related commensals of animals and humans, and two human pathogens, *Neisseria gonorrhoeae* and *Neisseria meningitidis*. The mechanisms allowing commensal *Neisseria* to maintain a niche in their host is little understood. To identify genes required for persistence, we screened a library of transposon mutants of *Neisseria musculi*, a commensal of wild-caught mice, in CAST/EiJ mice, which persistently and asymptomatically colonizes. Approximately 500 candidate host interaction genes were identified. A subset of these are homologs of *N*. *meningitidis* and *N*. *gonorrhoeae* genes known to modulate pathogen-host interactions *in vitro*. Many candidate genes have no known function, demonstrating how much remains to be learned about *N*. *musculi* niche maintenance. As many genes of unknown function are conserved in human adapted *Neisseria*, they provide a gateway for understanding *Neisseria* persistence mechanisms in general.

## Introduction

The microbiota is well documented to play a key role in human immune system development and maintenance, nutrient acquisition, and hormone production, among other processes [1]. How microbes/commensals engage the host to shape and maintain their own ecological niche is still not well understood. Dissecting the contribution of individual species to these processes faces considerable hurdles. Most members of the microbiota are not yet culturable, and of those that are, many are genetically intractable [2,3]. The tropism of some species for humans limits the ability of small animal models to accurately recapitulate microbe-human interactions, especially in the context of long-term carriage. To identify the mechanisms used by commensals to interact with their natural host, novel approaches are required.

The *Neisseria* genus provides this opportunity. This large group of Gram-negative β-Proteobacteria are commonly found in humans and animals [4]. *Neisseria sicca*, *N*. *subflava*, *N*. *flavescens* and *N*. *lactamica*, among others, inhabit the human oropharynx, establishing their niche early in human development [5–7]. *Neisseria spp*. have also been identified in a wide array of wild and domesticated animals [8].

The recent isolation of *Neisseria musculi* from a wild-caught mouse and its subsequent characterization opened an avenue of approach for studying commensal colonization [9–11]. *N*. *musculi* establishes long-term (at least one year) residence in the oral cavity and gut of susceptible strains of lab mice (see [9]) which do not naturally harbor *Neisseria*; the animals are healthy throughout. The *N*. *musculi* genome has been sequenced and annotated, and, like the other *Neisseria spp*. examined, the bacterium can be manipulated genetically [10,12]. The pairing of *N*. *musculi* with the mouse, its natural host, makes it possible to study commensal *Neisseria* niche development and maintenance, from the standpoint of both bacterium and host.

The *N*. *musculi*-mouse model can potentially inform on *Neisseria* pathogenesis as well. Two members of this genus have high pathogenic potential: *N*. *meningitidis* and *N*. *gonorrhoeae*, which respectively causes meningococcal meningitis and septicemia, and gonorrhea [13,14]. These species infect humans exclusively, and their tropism is dictated in part by their interactions specifically with human isoforms of transferrin, lactoferrin, IgA1 and immunoregulators CD46 and CEACAM [15–20]. Infection does not necessarily result in disease. *N*. *meningitidis* is a common inhabitant of the oropharynx; in healthy adult populations, *N*. *meningitidis* carriage rates can exceed 30% and asymptomatic colonization may last for many months [21]. Similarly, exposure of the urogenital tract, oropharynx, and rectum to *N*. *gonorrhoeae* does not automatically lead to symptomatic disease [13]. Between 50–70% of women with *N*. *gonorrhoeae* infections have no symptoms [22].

*N*. *meningitidis* and *N*. *gonorrhoeae* descended from a commensal *Neisseria* ancestor and are genetically related to present day commensal *Neisseria spp*. [23]. This shared ancestry with commensals suggests that their tendency to cause persistent asymptomatic infection may be governed by a shared repertoire of host interaction factors. Indeed, of the approximately 177 *N*. *gonorrhoeae* and *N*. *meningitidis* genes previously reported to encode host interaction factors, 69 are conserved in all 19 other *Neisseria spp*. evaluated [24]. While genes unique to *N*. *gonorrhoeae* and *N*. *meningitidis* undoubtedly contribute to pathogenesis, the importance of the shared host interaction factors to infection cannot be discounted. Moreover, these shared host interaction factors may also contribute to the ability of commensal *Neisseria* to sporadically cause disease [25].

Studies of *N*. *gonorrhoeae* and *N*. *meningitidis* host interaction factors largely rely on *in vitro* systems, and studies of inflammatory responses to infection depend on the use of genetically, surgically and/or chemically modified mice [26–28]. While these experimental systems have provided invaluable insight into early events in pathogen*-*host interactions, they cannot be used for studies of long-term carriage. The *N*. *musculi*-mouse model system answers this need.

To identify *N*. *musculi* genes putatively required for long-term colonization, we created a library of Tn5 transposon mutants and screened the library in CAST/EiJ mice for mutants that fail to persist in the oral cavity and gut. By comparing the inoculum library with those recovered from the two sites at various times after inoculation, we identified a list of *N*. *musculi* genes that are (presumed) required for long-term carriage in these niches. Several *N*. *gonorrhoeae* and *N*. *meningitidis* host interaction genes are on this list, confirming the *in vivo* importance of these pathogen homologues. Also on the list are genes for the biosynthesis of a capsular polysaccharide (cps), which is widely considered a virulence attribute of *N*. *meningitidis* and other pathogens. *In vivo* testing of a genetically defined *cps* deficient mutant confirmed a role for this "virulence" factor in long-term colonization by *N*. *musculi*.

## Materials and methods

### Ethics statement

Animal experiments were conducted in accordance with recommendations in the Guide for the Care and Use of Laboratory Animals of the National Institutes of Health. All experiments were approved by the University of Arizona Institutional Animal Care and Use Committee (PHS Animal Welfare assurance number A-3248-01) under protocol 10–203.

### Bacterial culture

*N*. *musculi* strain AP2365 was used for all experiments in this study (S1 Table) [10]. Bacteria were struck for single colony isolation on GCB plates containing Kellogg’s Supplement I & II and Rifampicin (Rif) (40 μg/mL) for maintenance, and Kanamycin (Km) (50 μg/mL) for selection of Tn5-containing clones. All *Neisseria* cells were grown at 37°C, 5% CO_2_.

### DNA extraction

Eighteen-hour lawns of *N*. *musculi* were collected into TES buffer (50 mM Tris pH 8, 20 mM EDTA, 50 mM NaCl) with 1% SDS and thoroughly resuspended by vortexing [29]. DNA was extracted from the suspension using two rounds of 25:24:1 phenol/chloroform/isoamyl alcohol purification followed by ethanol precipitation in sodium acetate (0.3M final concentration). RNA was removed with RNAseA (Qiagen) and DNA integrity was confirmed by gel electrophoresis.

### Construction of Tn5 library

DNA purified from *N*. *musculi* was modified using the EZ-TN5-KAN-2 Transposon Kit (Lucigen) with modifications to the manufacturer’s instructions [30]. 1.5 μg of DNA was incubated 2 hours at 37° C with 0.15 pmole of EZ-TN5 KAN-2 transposon, 1.5 units of Tn5 transposase and 1X reaction buffer (0.05 M Tris-acetate pH 7.5, 1.5 M potassium acetate, 10 mM magnesium acetate, 4 mM spermidine). The reaction was stopped with 0.1% SDS and heated for 20 minutes at 72° C prior to column purification (Clean and Concentrate Kit, Zymo). 1 unit of T4 DNA polymerase (Thermofisher) and 2 nmol of DNTPs (Thermofisher) were added to the heat-killed reactions and incubated another 20 minutes at 11° C to repair transposon-DNA junctions prior to column purification (Clean and Concentrate Kit, Zymo). Samples were eluted in Tris (5 mM, pH 8), and stored at -20° C until transformation. This procedure was repeated three times.

**Fig 1:**
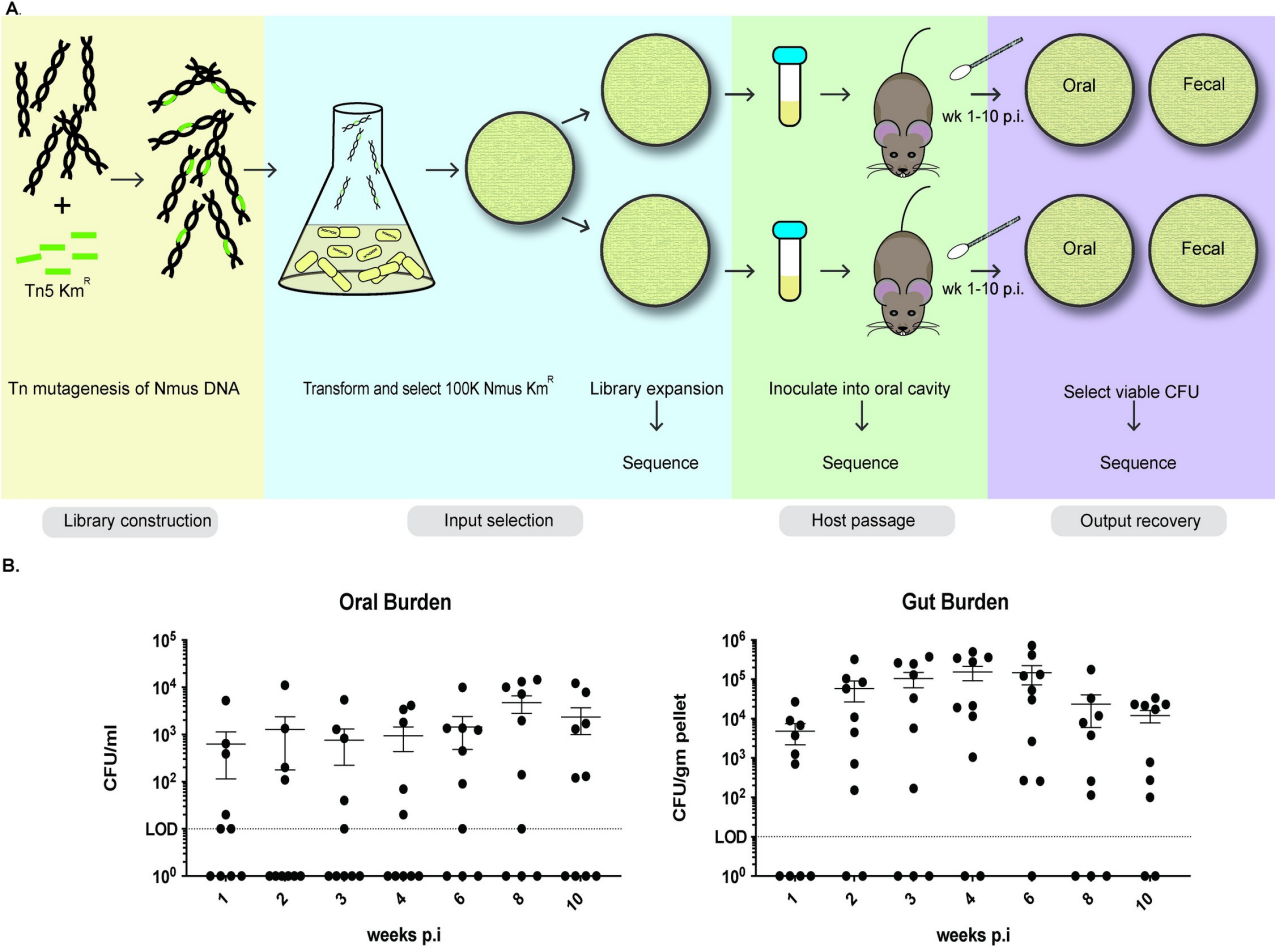
Construction and screening of the *N*. *musculi* Tn5 transposon library. A. Genomic DNA was extracted from *N*. *musculi* and transposed *in vitro* using a Tn5EZ-KM kit prior to transformation into Wt *N*. *musculi* and selection on Kanamycin. Mutagenesis and transformation were repeated until a total of approximately 100,000 CFU were isolated, at which point the libraries were replicated and frozen. For inoculation, a single library replicate per cohort of mice (n = 5) was cultured on selective media, and resuspended bacteria were introduced into the oral cavity of CAST/EiJ mice. Each cohort of mice was sampled weekly for the duration of the study, and bacteria were cultured from fecal pellets and oral swabs on selective media. Genomic DNA was isolated from the library replicate passage and the inoculum, and from bacteria recovered in fecal and oral samples. These samples were submitted to the University of Arizona Genetics Core for Tn5 junction enrichment, library preparation, and sequencing on an Illumina MiSeq instrument. B. Total bacterial burden recovered from library 1 and 2 cohorts. Data are expressed as per mouse CFU/ml total sample volume for oral isolates, CFU/gm fecal pellet for gut samples. Points appearing below the LOD indicate a mouse without a detectable burden; they are plotted to illustrate mouse group size. Nmus; *Neisseria musculi*.

For transformation, 18-hour lawns of *N*. *musculi* were collected in pre-warmed GCB with 10 mM MgSO_4_ and incubated 30 minutes with 1 μg of the *in vitro*-transposed DNA, spotted on a GCB plate and incubated an additional 8 hours at 37° C 5% CO_2_. Colonies were then harvested and selected on GCB agar containing Km (50 μg/mL) for 48 hours 37° C 5% CO_2_. This procedure was repeated 3 times to collect approximately 100,000 Km^R^ CFUs. Each plate was replicated 5 times onto supplemented GCB agar containing Km (50 μg/mL) and incubated until colonies were visible (~24–36 hours). Cells from replicate plates were pooled into single tubes containing 5 ml of GC broth with 20% glycerol to create single library copies, and frozen at -80°C. A summary of the transposon library construction and screening approach is found in Fig 1A.

### Animal inoculation and sample collection

All mice used in the study were obtained from the Jackson laboratory (Bar Harbor ME). Three days prior to inoculation of the mutant library into mice, one replicate of the library was thawed and plated onto supplemented GCB agar containing Km (50 μg/mL). After a 36-hour incubation, each plate was replicated onto a fresh agar plate, to ensure only viable bacteria were collected for inoculation, and incubated another 36 hours. On the day of inoculation, the replicated plates were collected in Dulbecco’s PBS, thoroughly vortexed and the OD_600_ was measured in a spectrophotometer. An aliquot of the stock was corrected to an OD_600_ = 2 or OD_600_ = 4 in sterile DPBS for inoculation; the remainder was processed for DNA extraction. The inoculum was introduced orally into 5 CAST/EiJ mice pre-screened for the absence of *Neisseria*, following established procedures [11]. An aliquot of the inoculum was plated on supplemented GCB agar to establish the dose, and the remainder was processed for DNA extraction. Every week for 10 weeks post-inoculation, the mice were sampled for CFU enumeration. Oral swabs were collected as previously described [11]. For each mouse, fecal samples were collected over a 30-minute period in a pre-sterilized canister lined with a sterile paper towel. Pellets from each mouse were collected in a single tube, weighed, then suspended in unsupplemented GC broth with 20% glycerol. A 100 μl aliquot of each sample was plated on supplemented GCB agar containing Rif (40 μg/mL) to assess bacterial burden; the remainder was banked at -80° C. To prepare samples for sequencing, the remainder of the sample was plated on supplemented GCB agar containing Rif (40 μg/mL) and Km (50 μg/mL) to select for Tn5-containing bacteria from each timepoint. The colonies on Rif/Kan plates were pooled by dose, cohort, and experiment, and DNA from each pool was extracted. This process was repeated using a second library replicate in 5 additional CAST/EiJ mice.

For validation of Tn5 mutants, Wt, Δ*cps228* and complemented strains were harvested in PBS as 18-hour lawns, and adjusted to an OD_600_ = 2 prior to oral inoculation in 6–8 week old A/J mice, which are also permissive for *N*. *musculi* colonization, according to established procedures [11]. Oral swabs and fecal pellets were collected each week for 8 weeks in GC broth with 20% glycerol for storage at -80° C, and aliquots were plated on supplemented GCB Rif (40 μg/mL). CFUs were enumerated, and bacterial burden normalized to total sample volume or CFU/gm fecal pellet for each strain.

### Next generation sequencing

Sequencing was performed at the University of Arizona Genetics Core following previously described methods [31]. Briefly, 2 μg of DNA was sheared to 500 bp by ultrasonication on a Covaris S2. Samples were end-repaired, A tailed and ligated to adaptors Ind_Ad_T and Ind_Ad_B by NEBnext DNA library prep kit. PCR enrichment of TN5 junctions was performed with primers Tn-FO and Adapt-RO using Kapa HotStart HiFi (Roche) using thermocycling conditions as follows; 95° C for 5 minutes, 94° C for 45 seconds, 56° C for 1 minute, 72° C for 1 minute for 22 cycles, and a final extension at 72° C for 10 minutes. Samples were purified using a MagBio HighPrep PCR cleanup kit (MagBio Genomics) and amplicons from 100 to 600 bp were captured using a 2% agarose cassette on a Blue Pippin instrument (Sage Science). Amplicons were indexed using a Nextera V2 dual index kit (Illumina) and purified by MagBio HighPrep PCR cleanup. Purified, indexed amplicons were then quantified on a Qbit fluorometer, and sequenced on an Illumina MiSeq instrument as 75 bp paired end reads. Sequencing was repeated on these libraries in 3 separate runs to generate enough reads for analysis, and the reads were merged by sample after initial demultiplexing.

### Transposon insertion sequencing analysis

Cutadapt was used to filter and trim adaptors and Tn5 sequences from reads, and pairs lacking Tn5 sequences or failing minimum length filters were discarded [32]. The remaining reads were aligned to the *N*. *musculi* reference genome, (accession CP06414.1 and CP06415.1, strain NW831/AP2031) with BWA-MEM [12,33]. The resulting files were further analyzed using tools from the Bio-Tradis pipeline [34]. Insertion plots were generated as described using the tradis_plot script. To remove multimapping reads, reads with a mapping quality less than 30 were discarded. Gene insertions, i.e., genes interrupted by Tn5, were identified using the tradis_gene_insert_sites script, and insertions in the 3’ 10% of the open reading frame (ORF) were ignored. Analysis was performed on samples merged by replicate, time point and sample site. Putative housekeeping genes were identified using the tradis_essentiality.R tool as previously described [34–36]. Briefly, insertion index thresholds were determined by log-odds ratios calculated on fits of gamma distributions of insertion indices in each sample. Putative housekeeping genes were identified as those with insertion indices below the calculated essentiality threshold in both replicates of the passage samples. Host interaction candidates were identified using tradis_comparison.R, comparing Tn5 derived reads in the inoculum to mouse output samples. An ORF was classified as a host interaction candidate gene using a cutoff of log_2_-fold change > [1] output vs. inoculum, and false discovery rate adjusted p value (q) < 0.05 according to previously described methods [37]. Repetitive transposase elements were removed from the analysis to preclude false positive assignment of essentiality, as these are present in multiple copies in the genome and as such any reads originating from these sites would have been marked as multi-mapping. Genes appearing in the housekeeping gene set were ignored in further analysis of host interaction candidate genes due to their low initial saturation in an effort to improve the stringency of our screen. To identify pathways involved in host interaction, pathway enrichment analysis of fold-change ranked significant hits with the enrichKEGG tool from clusterProfiler Bioconductor package implemented in R [38]. To expand the number of genes mapped to pathways, analyses were conducted using a minimum gene set size of 1, and a Benjamin-Hochberg adjusted *p* value cutoff of 1.0. An adjusted *p* value of 0.1 was used to assign significance. The *N*. *musculi* functional annotation hosted on KEGG was used as the reference for these analyses [39]. COG assignments were made using eggnog-mapper V2 with protein sequences from the AP2031 reference [40]. Phage annotation was performed using the PHASTER server on the *N*. *musculi* reference genome [41]. Comparisons of *N*. *musculi* host interaction candidates to genes encoded by the rest of the *Neisseria* genus were performed using KEGG Sequence Similarity Database (SSDB) Gene Function Identification Tool (GFIT) Smith-Waterman searches, blastP, and blastN [39]. Heatmap clustering and visualization was performed using the complexHeatmap Bioconductor package [42].

### Validation of capsular polysaccharide mutants

The noncoding 228 bp DNA segment between the capsular synthesis and transport regions in *N*. *musculi* was deleted and replaced with a Kanamycin (Km) cassette [43]. A DNA fragment (IM132, S1 Table) containing a neomycin phosphotransferase gene and sequences flanking *csiA/A1* and *ctrA* was ligated into pGEM-T (Promega) and the ligation product was confirmed by restriction digestion and sequencing. The recombinant plasmid was transformed into Wt *N*. *musculi* AP2365 and transformants were selected on supplemented GCB agar containing Km (50 μg/ml). Km resistant mutants were confirmed by Sanger sequencing of PCR amplicons generated by primers IM059 and IM060 (S1 Table). To construct the complemented strain, the intergenic region of one capsule nonproducing mutant, Δ*cps228*, was transformed with a PCR product generated from Wt DNA using primers IM133 and IM134 (S1 Table).

### Analysis of *cps* transcripts

Wt *N*. *musculi* AP2365, Δ*cps228* and complement strains were grown to mid-log phase in supplemented GC broth at 37° C 5% CO_2_ without shaking. Total RNA was extracted using TriZol (Invitrogen) according to the manufacturer’s recommendations, and treated with DNase (DNA-free, Ambion) to remove chromosomal DNA. RNA was quantified using a Nanodrop (Thermo Scientific), and 1 μg of RNA was used in first strand synthesis using M-MLV reverse transcriptase (Promega) according to the manufacturer’s instructions. PCR to characterize transcripts was conducted using GoTaq green master mix (Promega) with primers to amplify transport, translocation and capsule synthesis transcripts and junctions between each putative transcript (S1 Table). 16S and genomic DNA (gDNA) controls were amplified from total RNA.

### Growth curves

Wt AP2365, Δ*cps228* and complemented strains were harvested from 16-hour lawns grown on supplemented GCB agar and adjusted to an OD_600_ = 0.05 in 2 ml GC broth with 1% Kellogg’s supplement 1 and 0.1% supplement II in 50 mm dishes. Samples were incubated at 37° C 5% CO_2_ for 8 hours, and OD_600_ was measured every 2 hours on a Beckman coulter spectrophotometer. The absorbance at each time point was normalized to the density of that sample at t = 0.

### Capsule extraction and staining

To examine capsule production, 16-hour lawns of Wt *N*. *musculi* and capsule mutant strains, *N*. *gonorrhoeae* MS11 and *N*. *meningitidis* FAM18 cells were harvested in PBS and adjusted to an OD_600_ = 1.0. Cell suspensions were incubated at 55° C for 30 minutes and pelleted. Supernates were filtered using an Amicon Ultra 10k NNWL centrifugal filter device (Millipore) and concentrated 10x. 30 μl of concentrate was run on a 7.5% SDS PAGE gel and stained overnight with 0.125% Alcian blue (Sigma). The gel was destained in 40% ethanol 5% glacial acetic acid until all background was removed and imaged on a LiCor Odyssey instrument.

### Statistical analysis

Normalization and statistical analysis of transposon insertion data was completed using methods implemented by the Bio-Tradis tool set. These methods have been extensively described elsewhere [34–36]. Pathway enrichment statistical analysis was conducted in R using the clusterProfiler package. Multiple testing was corrected using the Benjamin Hochberg correction for both Bio-Tradis and clusterProfiler analyses. Pearson correlation analysis was used to compare gene insertion indices and read counts from each host- passage sample replicate. Comparison of COGs between the housekeeping candidate pool and host interaction candidate pool was performed by Two-sided Fisher’s exact test. Mouse burdens were tested for normality using the Kolmogorov-Smirnov and Shapiro-Wilks tests, and analyzed using Kruskal-Wallis tests with Dunn’s comparison to Wt controls for each strain. Correlation analysis, COG comparisons, and mouse data analysis was conducted in GraphPad Prism V9. All *in vitro* experiments were performed in 3 biological and 3 technical replicates.

## Results

### Screening of a library of Tn5 *N*. *musculi* mutants in mice for genes required for long-term colonization

We constructed and screened a library of Tn5 mutants of *N*. *musculi* in CAST/EiJ mice to identify mutants in the inoculum that were absent or underrepresented in oral cavity and gut samples at various times post-inoculation. Each of 2 replicates of this library was inoculated into 5 CAST/EiJ mice (approximately 1x10^7 mutants per inoculum) (Fig 1A and 1B). *N*. *musculi* burden in the oral cavity and gut were determined weekly for 10 weeks. At week 4, *N*. *musculi* CFUs were recovered in large numbers from the oral cavity and gut of 50% and 80% of the mice, respectively. By 6 and 8 weeks, *N*. *musculi* was recovered from 70% of the mice. The number of mice with detectable *N*. *musculi* CFUs in the oral cavity increased over time, while *N*. *musculi* CFUs recovered from fecal samples remained relatively stable between 4 and 6 weeks.

*N*. *musculi* DNA was isolated from the inoculum generated from each library as well as from the initial culture from which the inoculum was derived. Tn5 junction enrichment and high throughput sequencing of Tn5 insertion sites was performed at the University of Arizona Genetics Core. A total of ~62 million Tn5-directed reads were synthesized from all samples; over 90% of mate 1 reads contained the Tn5 adaptor. An average of 75% of the Tn5-containing read pairs in each sample mapped to the *N*. *musculi* chromosome and plasmid. These reads were derived from 327633 unique Tn5 insertions in the library 1 inoculum and 304165 in the library 2 inoculum. This represents a transposon insertion every 9 bp, allowing for identification of host interaction candidates after passage through the mouse. Identification and analysis of Tn5 insertions was performed using tools from the Bio-Tradis pipeline.

To identify housekeeping genes and genes with low Tn5 saturation, the Bio-Tradis essentiality.R tool was used to analyze sequences from library 1 and 2 initial culture samples. Analysis of distributions of gene insertion indices of these samples initially identified ~1100 candidates as possibly required for *in vitro* growth with agreement between libraries (S1A Fig). These genes were further curated to remove repetitive transposable elements; this avoids genes being erroneously classified as essential due to a lack of insertions as multimapping reads were discarded during the analysis step. The curation yielded a final set of 776 putative housekeeping genes with a lower than anticipated gene insertion index and 192 genes without detectable insertions in library passage and inoculum samples (S1 Data). Annotation, pathway mapping and over representation analysis revealed their functions were predominately associated with central and secondary metabolism, ribosomal structure and biogenesis, cell envelope biosynthesis, cell division, and nutrient acquisition and transport (S1B and S1C Fig). The prevalence of genes associated with vital physiologic processes, including those known to be essential in other organisms, (*e*.*g*., signal peptidase 1, ribosomal proteins, NrdB, elongation factor Tu) supports our notion that insertions in these open reading frames convey a defect in *in vitro* growth [44].

### Identification of *N*. *musculi* genes required for long-term colonization

To focus on genes important for persistence in the mouse, gut samples from week 6 and gut and oral samples from week 8 were further analyzed. We recovered a total of ~2.6 x 10^6 *N*. *musculi* CFU/gm fecal pellet in week 6, while week 8 gut and oral samples contained a total of ~2.7 x10^5 CFU/gm fecal pellet and ~4.7 x 10^4 CFU/ml sample, respectively. Bacteria isolated from these samples were pooled by library replicate, dose, time point and sample site, and DNA was extracted and sent for Tn5 insertion sequencing.

**Fig 2:**
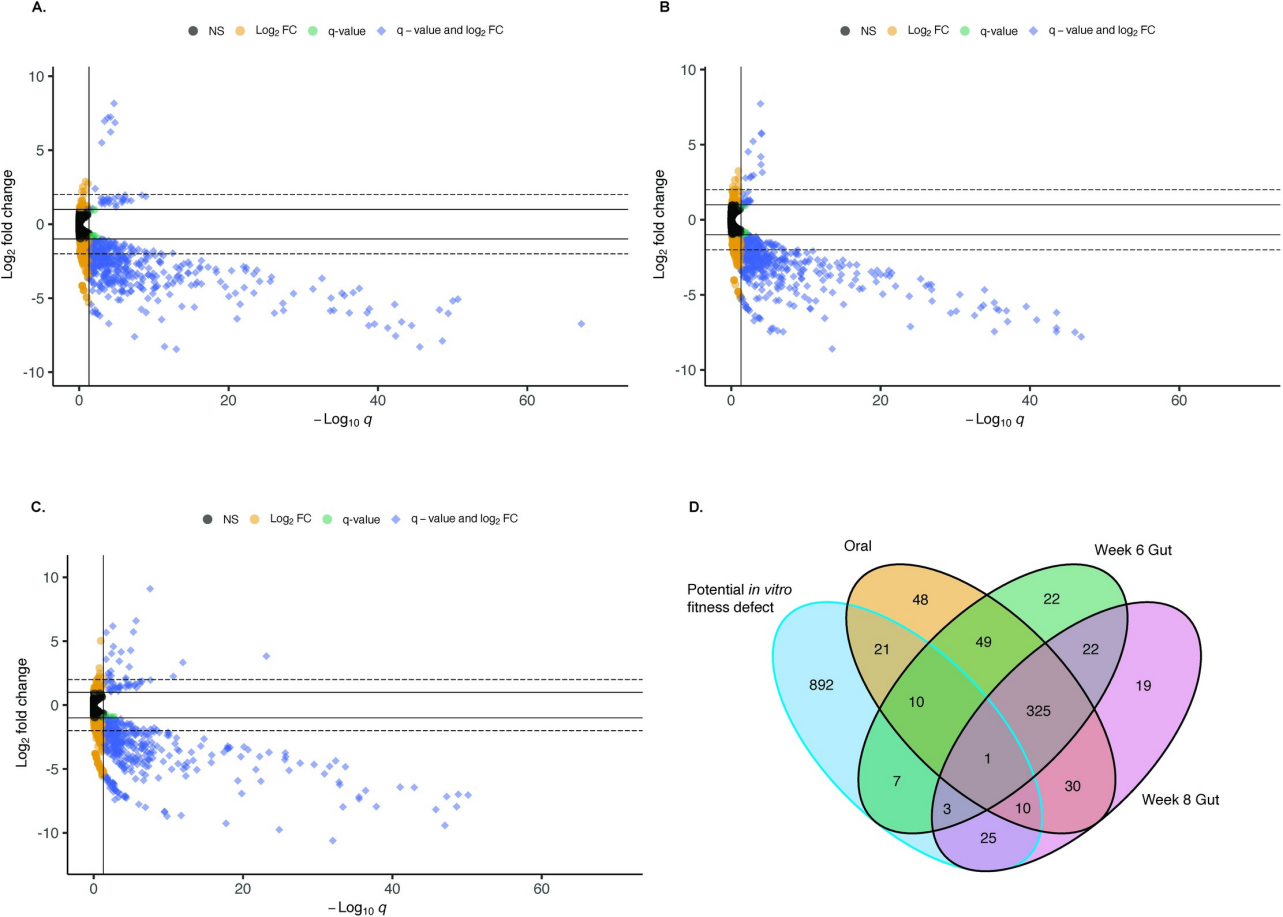
Identification of host interaction candidate genes. Volcano plots depict the results of EdgeR analysis of Tn5 directed read counts in Oral Week 8 samples vs. Inoculum (A), Gut Week 6 samples vs. Inoculum (B), and Gut Week 8 samples vs. Inoculum (C). Each ORF is plotted to show log_2_ Fold Change (FC) vs. the false discovery rate adjusted P value (q). Significance cutoffs (q = 0.05 and Log_2_ FC = 1 & -1) are depicted with solid lines. Dashed lines represent Log_2_ FC = 2 & -2 for comparison. Genes passing fold change and q value significance cutoffs are shown in blue. A total of 592 genes met these significance criteria. D. Comparison of genes in host interaction and housekeeping candidate pools meeting significance criteria. Candidate host interaction genes overlapping with the candidate housekeeping gene set fall within the blue ellipse. A total of 77 genes appearing in this pool were excluded from analysis of host interaction candidates.

To identify host interaction genes, defined as insertion mutants in the inoculum that are underrepresented in the mouse-passed samples, Tn-directed reads from oral and gut samples were compared to those from the inoculum, using tools from the Bio-Tradis pipeline. This identified a total of 592 ORFs which met our criteria for significance in the mouse-passaged samples (q < 0.05 and an absolute Log_2_ Fold Change of >1 compared to inoculum; Fig 2A and 2C). Seventy-seven ORFs from week 6 and 8 samples were also present in the housekeeping candidate pool. These were removed from further analysis, as were repetitive transposable elements (Fig 2D).

Of the remaining 515 candidates with significantly altered abundance in comparison to the inoculum, insertion mutants in 473 of these ORFs were depleted, while 42 were enriched. Insertions in 325 ORFs had significantly altered abundance in all 3 host output samples, 79 had altered abundances in the oral cavity and one gut sample, 48 were unique to the oral cavity, and 64 were unique to the gut (Fig 2D and S2 Data). On average, insertions in 70% of these genes displayed an absolute fold-change of 2 compared to the inoculum.

**Fig 3:**
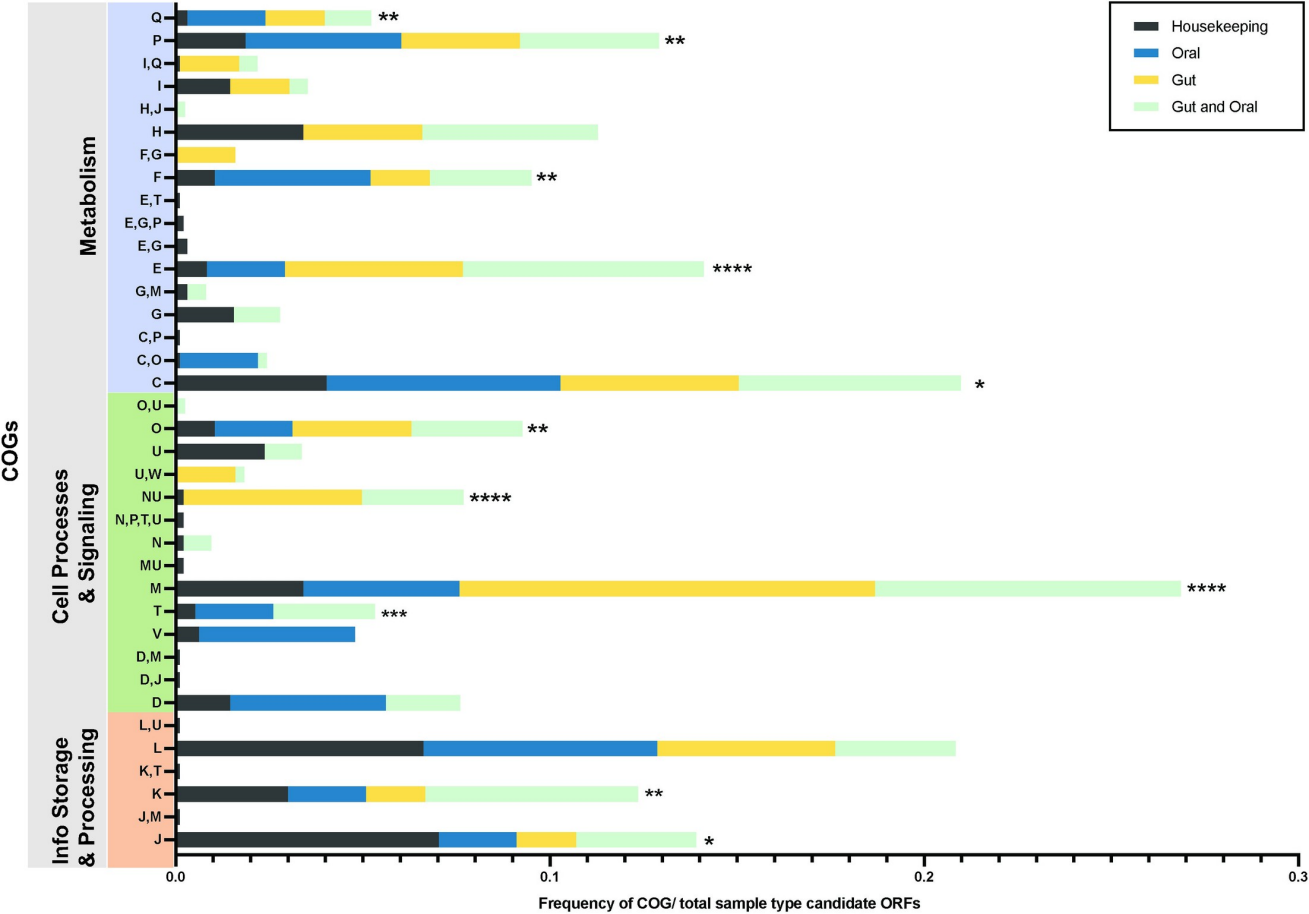
Function-based classification of candidate genes. COG category classification and frequency of candidate genes identified either as required for *in vitro* growth (housekeeping) or significantly altered in abundance in host passage samples (Gut, Gut and Oral, Oral). Two-sided Fisher’s exact test was used to compare frequency of COGs in the housekeeping pool to their total host site frequency. COG category J was significantly more prevalent in the housekeeping pool, while categories Q,P,F,E,C,O,NU, M,T and K were more prevalent in host sites. Classification was conducted using eggNOG mapper v-2. Bar color indicates sample type. * = *p <* 0.05, ** = p < 0.01, *** = p < 0.001, **** = *p <* 0.0001. Category definitions are based on the Database of Clusters of Orthologous Genes as follows: J, Translation, Ribosomal Structure and Biogenesis; K, Transcription; L, Replication, Recombination and Repair; B, Chromatin Structure and Dynamics; D, Cell cycle control, cell division and chromosome partitioning; V, Defense Mechanisms; T, Signal Transduction Mechanisms; M, Cell Wall/Membrane/Envelope Biogenesis; N, Cell Motility; W, Extracellular Structures; U, Intracellular Trafficking, Secretion, and Vesicular Transport; O, Posttranslational Modification, Protein Turnover, Chaperones; C, Energy Production and Conversion; G, Carbohydrate Transport and Metabolism; E, Amino Acid Transport and Metabolism; F, Nucleotide Transport and Metabolism; H, Coenzyme Transport and Metabolism; I, Lipid Transport and Metabolism; P, Inorganic Ion Transport and Metabolism; Q, Secondary Metabolite Biosynthesis, Transport and Catabolism. Genes without an assigned COG or in category S (function unknown) were omitted from this analysis.

Based on COG annotation, a large proportion of candidate host interaction genes functioned in cell envelope biogenesis, motility, amino acid transport and signal transduction (Fig 3). Enrichment analysis of 325 candidate genes required at each sample site and time point corroborated the COG functional annotation; pathways associated with amino acid and secondary metabolite biosynthesis and bacterial chemotaxis were over-represented in this category (S2A and S2B Fig). Of note, a large number of candidates are predicted to function in branched chain amino acid degradation, the citrate cycle and gluconeogenesis. This may suggest that mutants lacking the ability to utilize host amino acids are unable to overcome glucose limitation in the host, as observed in other organisms [45].

Of potential interest, a large proportion of the candidate genes have no assigned function, and 20% were annotated as encoding hypothetical proteins or encoding a protein with a domain of unknown function.

### *N*. *musculi* genes required for long-term colonization are conserved in human adapted *Neisseria*

**Fig 4:**
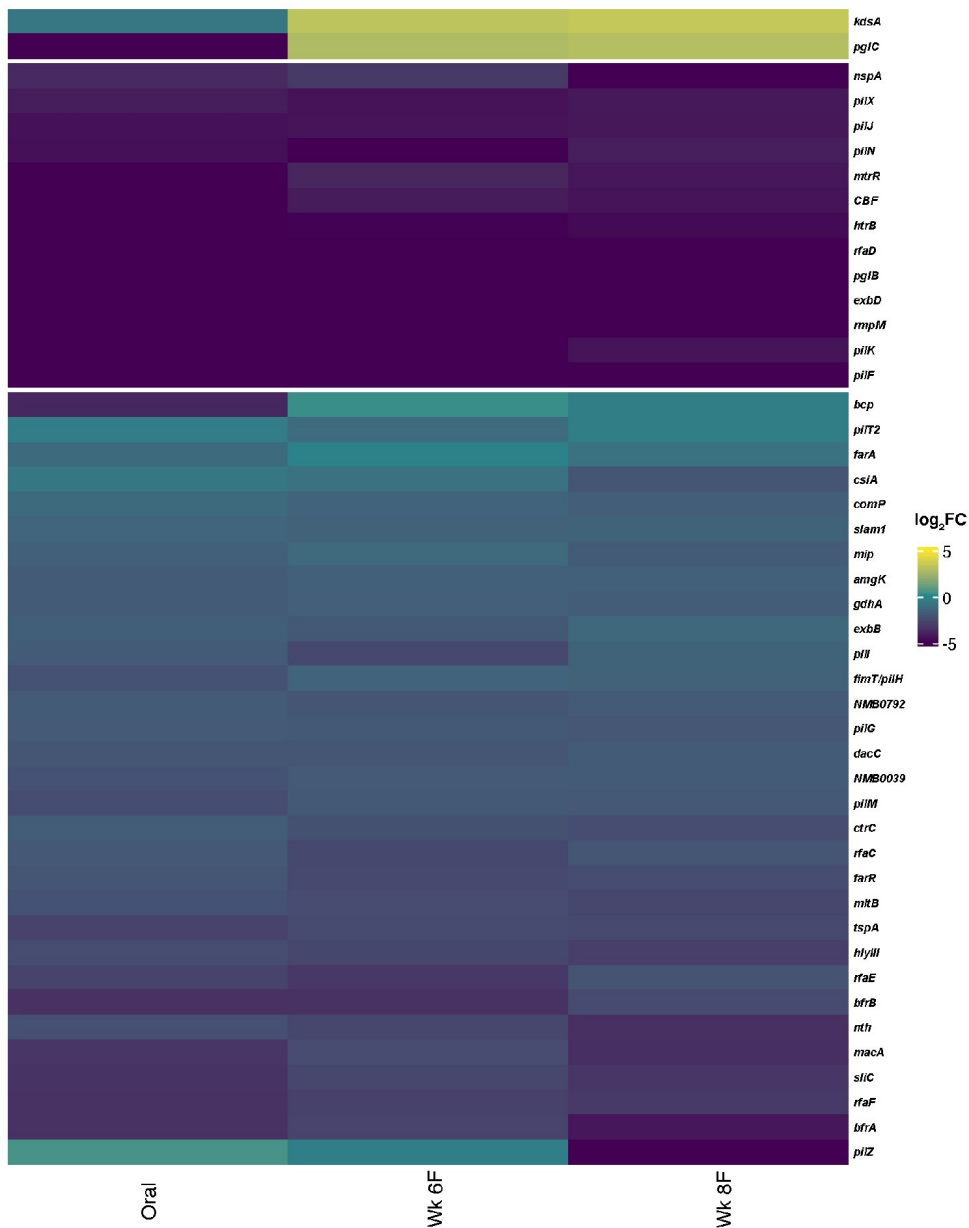
Homologs of human adapted *Neisseria* host interaction factors are required for *N*. *musculi* survival in mice. Relative abundance of *N*. *musculi* homologs of *Neisseria* host interaction factors identified in the *in vivo* screen. Heatmap depicts ORF Tn5 directed log_2_ fold change vs inoculum in *N*. *musculi* oral and gut samples. Genes are grouped by k-means clustering after calculation of Euclidian distances by complexHeatmap suite default settings. Correlation of gene name to *N*. *musculi* locus tag, protein similarity to human adapted *Neisseria*, and fold change data is found in S3 Data.

A large collection of genes, termed virulence genes, has been shown to influence the interactions of pathogens *N*. *gonorrhoeae* and *N*. *meningitidis* with human tissues and cells [23,46–53]. Many of these were subsequently discovered in commensal *Neisseria* [24,54]. Curation of 186 virulence genes and 50 genes with significant changes in expression during *in vitro* infection identified 160 *N*. *musculi* homologs; of these, 45 were identified by our Tn5 screen as required for long-term mouse colonization (Fig 4 and S3 Data). They function in LOS synthesis (*rfaCDEF*, *kdsA*), iron acquisition and detoxification (*bfrAB*, hemolysin III, *bcp*), efflux (*macA*, *farA*), transcriptional regulation (*farR*,), and cell wall recycling, synthesis and repair (*ltgD*/*mltB*, *dacC*), among others [55–65]. At least three ORFs, (*mip*, *nspA*, *cbf)*, encode homologs of surface expressed immunomodulatory proteins [60,66–68]. A transporter of surface associated proteins (*slam1*) was also required for persistence [69,70].

Our screen also identified many Type IV pilus (Tfp) genes. They encode the pilin assembly ATPase (*pilF*), minor pilins (*pilH*, *I*, *J*, *K* and *comP*), the Tfp assembly complex (*pilM*, *N*, *G*) and pilin glycosylation enzymes (*pglB*, *pglC*) [71–79]. *pilZ*, whose role in Tfp biogenesis/function has yet to be fully characterized [80], was significantly underrepresented only in the week 8 mouse-passaged gut sample. Proper elongation of the Tfp fiber requires peptidoglycan remodeling. In this context, it is interesting to note that a homolog of *dacC*, encoding a serine type D-ala-D-ala carboxypeptidase, was identified in our screen [65].

### *In vivo* screen identifies novel *Neisseria* host interaction factors

A closer examination of candidate genes not previously identified as *Neisseria* host interaction factors reveals a high degree of amino acid similarity of their protein products to those in human-adapted *Neisseria spp*. (S2 Table). These genes cover a broad range of functional categories, including cell envelope synthesis and modification, transcriptional regulation, signal transduction and transport. The prevalence of transport genes in this group highlights the need for agile metabolic adaption in response to nutrient limitation imposed by the host. Of interest are *cstA* (H7A79_RS05505) and *gluP* (H7A79_RS03010), both of which are encoded by human- and animal-adapted *Neisseria* and implicated in colonization and virulence in zoonotic bacteria. *cstA* encodes peptide transporter carbon starvation protein A, similar to *Campylobacter jejunii* CstA (62% identity over 688 amino acids). In this pathogen, the transporter is required for utilization of di- and tri-peptides as nitrogen sources under energy limiting conditions, implicating amino acid utilization as a requirement for host colonization [81]. *gluP* encodes a glucose/galactose MFS family transporter with similarity to *Brucella abortus* GluP (54.5% identity over 400 amino acids). While *gluP* has been identified as differentially expressed in *N*. *meningitidis in vitro* infection studies, its exact contribution to persistent carriage remains unknown in this context [44,82]. *B*. *abortus gluP* is essential for glucose uptake in host environments, as mutants display reduced survival in alternatively activated macrophages and cannot persist in a chronic mouse infection model [83,84]. These observations suggests that *N*. *musculi gluP* and *cstA* may function in a similar manner to allow long-term colonization.

**Fig 5:**
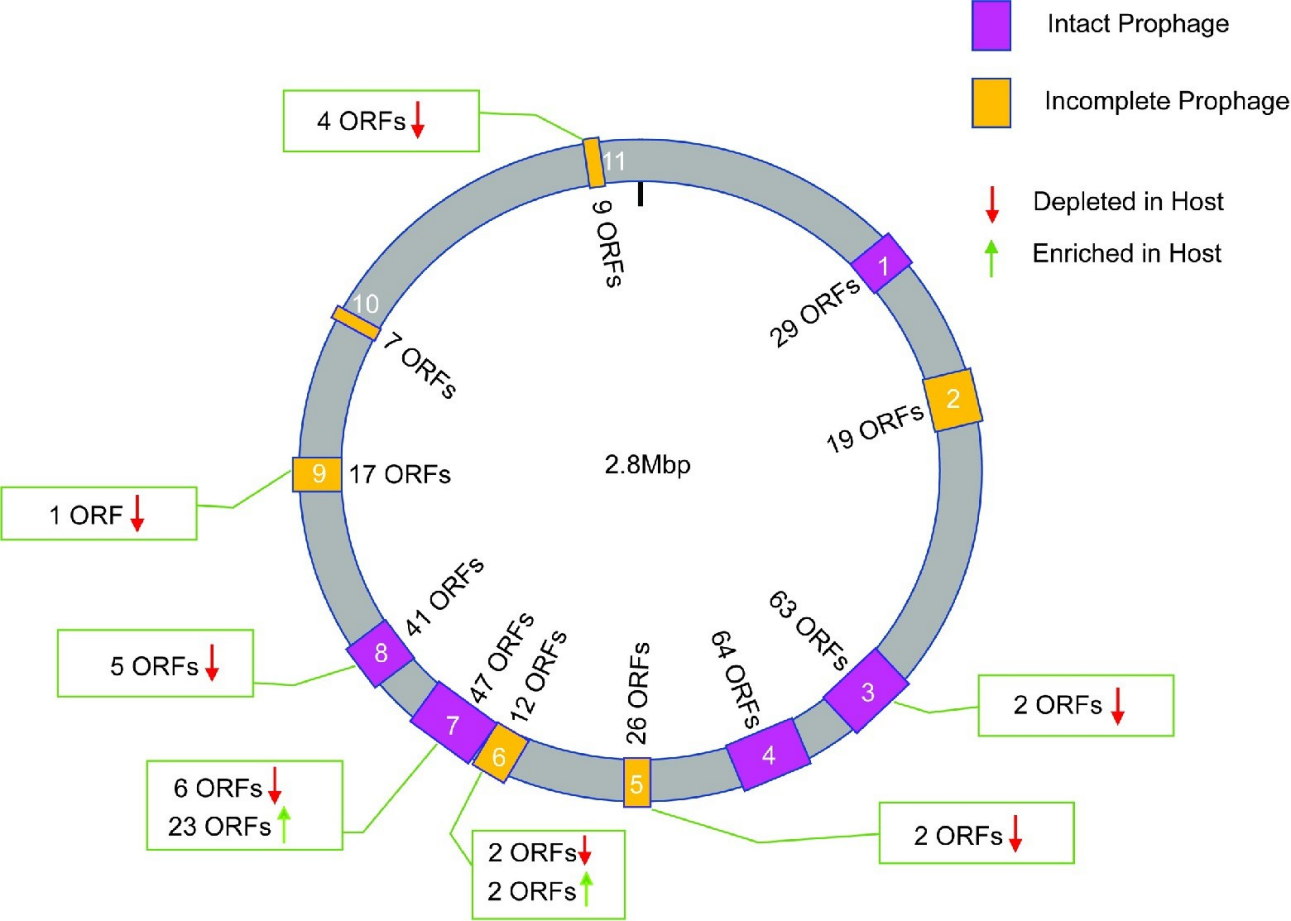
Identification of host essential candidate genes within putative prophage regions. PHASTER annotation of the *N*. *musculi* genome identified 11 regions associated with phage or phage like elements. Purple boxes indicate intact prophage regions while orange boxes indicate incomplete prophage regions as designated by PHASTER scoring algorithms. Arrows indicate abundance of insertion mutants after host passage in comparison to the inoculum. ORFs passing significance cutoffs after analysis are listed in green boxes for each prophage region.

Many host interaction candidates identified by our screen are phage related. Analysis of the *N*. *musculi* genome using the PHASTER annotation server identified 11 different phage-associated loci, 5 of which were considered complete prophages (Fig 5 and S4 Data). Twenty-two genes in these regions were depleted in host passage samples. Notably, ORFs in prophage region 7 in the host interaction candidate pool tended to be associated with nucleotide modification (when function could be assigned). These included H7A79_RS08530, encoding a DNA-methyltransferase; H7A79_RS08575, encoding an HNH endonuclease; and H7A79_RS08695, encoding putative dCTP deaminase similar to NMB0849 encoded by *N*. *meningitidis* MC58. The remaining phage-associated host essential candidates were located within regions 3, 5, 6, 9 and 11. Of interest in these clusters are homologs encoding the cold shock protein (c*spA*) and SsrA binding protein (*smpB*), which may be involved in post-transcriptional regulation of stress responses, in region 11; and a GpeE family phage tail chaperone protein, in region 3. Half of the phage associated host interaction candidates we identified have high similarity to ORFs encoded by pathogenic *Neisseria* (S4A Data). However, comparison of the candidate pool to genes encoded within *Neisseria* Nf1-4 prophages and MuMenB did not identify any candidates with high similarity to these ORFs (S4D Data) [85–87]. Common to all phage regions containing host interaction candidates were the prevalence of hypothetical ORFs that were likely bacterial in origin. Nine of 22 host interaction candidates in these regions had no assigned function and 8 lacked similarity to a phage gene product. This underscores the potential role of phage-mediated horizontal gene transfer in transporting host interaction factors between bacteria occupying a similar niche.

### Abolishing capsular polysaccharide (cps) expression negatively affects *N*. *musculi* persistence

Four genes annotated as cps components were underrepresented in our mouse-passaged samples compared to the inoculum. Although the capsule is widely considered a virulence factor, *cps* loci were recently reported in commensal *Neisseria* including *N*. *musculi* [9,88]. The identification of *N*. *musculi cps* genes in our Tn5 screen provides an opportunity to test the importance of this extracellular component to commensal colonization.

**Fig 6:**
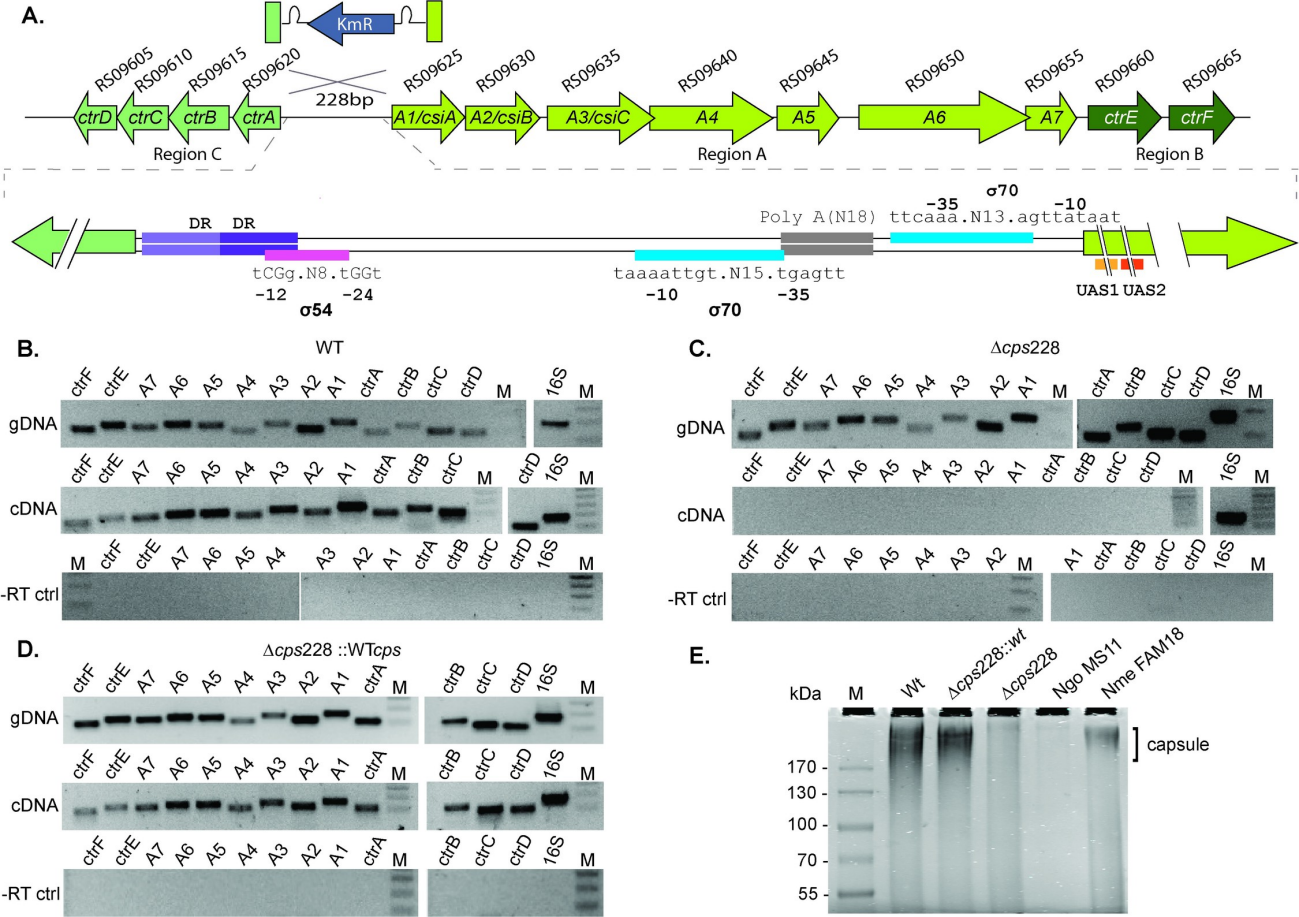
*N*. *musculi* mutants with a deletion of the capsule cluster regulatory region are unencapsulated. Validation of the candidate genes implicated in capsule biosynthesis and transport. A. Arrangement of the *N*. *musculi* capsule biosynthesis and transport genes. Shaded boxes indicate *Neisseria* genus capsule locus. Genes in Region C (transport), Region A (biosynthesis) and Region B (translocation) are given the name of the closest *N*. *meningitidis* homolog or functional analog, and the *N*. *musculi* GenBank refseq locus tag. Orange lines indicate the position of the upstream activator sequences (UAS1, tgtttttttgcacggcaca, and UAS2, tgtcaatgtcagagcact) in *csiA/*A1. The sequence of the 228 bp intergenic region between *ctr*A and A1 is highlighted by a dashed line. Sigma 70 binding sites are highlighted by a blue line, and Sigma 54 binding site is indicated by the pink line. The 17 bp direct repeat (DR, acaaatagccaaaaaca) is marked by a purple line. The grey line shows the location of the poly A tract. To test the hypothesis that the intergenic region between *A1* and *ctrA* control capsulation, the 228 bp segment of DNA was replaced with a Kanamycin cassette (blue arrow). B-D. Deletion of the capsule locus intergenic region ablates transcription of all capsule biosynthetic and transport genes. cDNA was generated from Wt (B), *cps* mutant (C) and the complemented strain (D), and primers specific to each gene were used to amplify transcript co-transcribed from the promoter located in the 228 bp intergenic region. No difference was observed in Wt or Δ*cps228*::Wt strains in comparison to genomic DNA controls and -RT controls. E. Loss of the capsule locus intergenic region results in loss of capsule production. SDS PAGE gel of capsule extracts isolated from *N*. *musculi* Wt, capsule mutant and complemented strains, compared to the *N*. *gonorrhoeae* MS11 and *N*. *meningitidis* FAM18 negative and positive controls, respectively. Alcian blue staining shows a reduction in polysaccharide content between the Wt and capsule deletion mutant extracts. M, molecular weight marker.

*N*. *musculi cps* genes (Fig 6A) are analogous to those in *N*. *meningitidis* with assigned functions in capsule synthesis (Region A), transport (Region B), and translocation (Region C) [89]. The organization and gene content of Region C and B are identical to that of other capsule expressing *Neisseria* species. In contrast, Region A is more divergent, although the annotated functions of the genes in this locus are similar to those in the analogous region in *N*. *meningitidis* serogroup I and other animal *Neisseria* [88,89].

The 4 *N*. *musculi cps* genes underrepresented in mouse-passaged samples are situated in Region C (H7A79_RS9610, encoding an ABC transporter permease homolog of CtrC) and Region A (H7A79_RS9625, encoding a CsiA homolog annotated as a UDP-N-acetylglucosamine epimerase (A1); H7A79_RS09645, encoding a putative rhamnosyl transferase (A5), and H7A79_RS09655, encoding a putative glycosyltransferase (A7) (S2 Table).

In Region C and A, start and stop codons of adjacent ORFs are often close together and in some cases overlap. The regulatory sequences for Regions C and A are likely located in a 228 bp segment of DNA between the two gene clusters; this intergenic region contains putative Sigma54 and Sigma70 consensus sequences, an Npa upstream activator sequence, as well as a 17-bp direct repeat and a 20-bp poly-Thymine tract, either or both of which may also serve as regulatory sequences (Fig 6A).

To determine whether the 228 bp intergenic region regulates expression of the capsule clusters, RT-PCR was performed on RNA isolated from *N*. *musculi* AP2365, using different combinations of primers spanning the junctions of consecutive ORFs. The genes in Region A (*csiA/A1*-*A7*) and Region B (*ctrE*, *ctrF*) are transcribed as a single mRNA. Genes in Region C (*ctrA*,*B*,*C*,*D*) are divergently transcribed, also as a single mRNA. This strongly suggest that transcription of the two operons is likely to be controlled from the 228 bp *csiA/A1*-*ctrA* intergenic region (S3 Fig).

To validate the requirement for capsule during long-term mouse colonization, the 228 bp intergenic region in Wt *N*. *musculi* was replaced with a Kanamycin cassette, generating Δ*cps*228. As predicted, deletion of this putative promoter region resulted in loss of capsule production in the mutant (Fig 6B–6D). RT-PCR experiments using RNA from Wt *N*. *musculi*, Δ*cps*228 and complemented strains demonstrated that loss of the *csiA/A1*-*ctrA* intergenic region ablated transcription of all genes in Regions A, B and C. Compared to Wt, complement and *N*. *meningitidis* FAM18 controls, extracts from Δ*cps*228 did not react with the capsule stain Alcian Blue (Fig 6E). Loss of capsule did not affect the *in vitro* growth rate of the mutant in comparison to Wt and complemented controls (S4 Fig).

**Fig 7:**
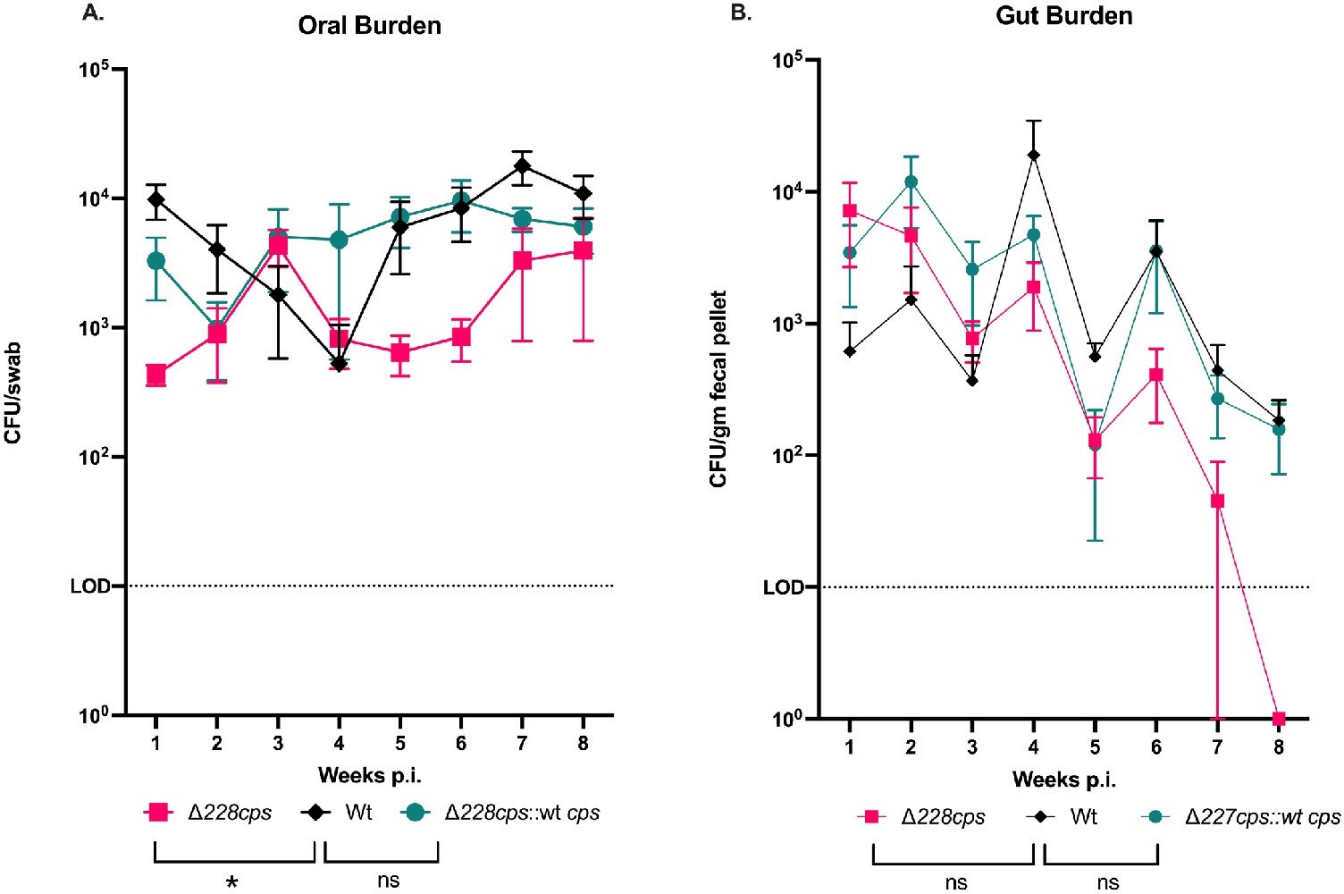
Loss of capsule significantly reduces *N*. *musculi* bacterial burden in the mouse oral cavity but not the gut during persistent colonization. A/J mice were orally inoculated with either Wt, Δ*228cps* or complemented strains (n = 5 per group). Oral swabs and fecal pellets were plated on selective agar at weekly intervals post inoculation to recover viable *N*. *musculi*. Total oral bacterial burden is significantly decreased in animals inoculated with the Δ*228cps* mutant in comparison to Wt controls (Kruskal Wallis test with Dunn’s comparison, * *p* = 0.0178, comparisons denoted by brackets in the legend), while no significant difference is observed in samples isolated from the gut. Bacterial burden is normalized to CFU/ml total sample volume for oral samples, and CFU/gm fecal pellet for gut samples. Points indicate mean burden by group with bars representing SEM.

To test the role of cps in host interaction, Wt *N*. *musculi*, Δ*cps*228, and complemented strains were inoculated into A/J mice. This mouse strain was used to ensure that the *cps* mutant phenotype was independent of any factor(s) associated with mouse strain background. *N*. *musculi* burdens from oral and fecal samples were assessed weekly for 8 weeks (Fig 7). Initially, no difference in absolute colonization frequency was observed between Wt, mutant and complemented control groups. However, starting 5 weeks post-inoculation, oral cavity burdens of Δ*cps*228 began to decline in comparison to the Wt and complemented strains. Mutant oral cavity burden remained lower than Wt, resulting in a significant difference in Δ*cps*228 oral burden in comparison to Wt controls over the entire study (*p* = 0.0178, Kruskal-Wallis test with Dunn’s comparison). This trend was replicated in gut burdens, although there was no significant difference in total gut burden between Wt and Δ*cps*228. Together, these results strongly suggest that unencapsulated *N*. *musculi* is able to colonize the oral cavity of mice, but is at a significant disadvantage in long-term colonization.

## Discussion

It is well established that commensal *Neisseria* are abundant members of the human microbiome, that *Neisseria* species are genetically related, and that pathogens *N*. *meningitidis* and *N*. *gonorrhoeae* establish asymptomatic infections in humans. Little, however, is known about how members of this genus maintain a long-term relationship with the host. Small animal models and *ex vivo* systems for characterizing *N*. *meningitidis*- and *N*. *gonorrhoeae*-host interactions limit studies to early events in infection and are unsuitable for examining a key facet of *Neisseria* behavior: persistence. Here, we report on the identification of genes in the mouse commensal *Neisseria musculi* that are critical for its ability to establish long-term colonization in its natural host.

We identified ~500 candidates involved in *N*. *musculi* colonization of CAST/EiJ mice for up to 8 weeks post-inoculation. Approximately 65% of these are required for persistence in both the oral cavity and gut. They span almost every functional category of cellular processes according to COG annotation and curated KEGG pathway analysis, although cell envelope biogenesis, amino acid metabolism, energy conversion, and motility are most common. Twenty-two candidates encode transcriptional regulators, suggesting tight control of expression of multiple pathways is required for niche maintenance in host environments. Of particular interest is the site specificity shown by some essential gene candidates: 48 were significant only in the oral cavity, while 63 were significant only in gut samples (S2 Data). No significant difference was seen in COG category frequency between these two sites (S5 Fig), however, it should be noted that the functions of many of these genes may be niche specific. For example, mutants in H7A79_RS01980, encoding a homolog of bacterioferritin comigratory protein (Bcp) were significantly depleted in oral samples but not the gut. Bcp functions as a hydroxyperoxide peroxidase involved in oxidative stress resistance [64,90]. Inactivation of H7A79_RS01980 may impede resistance to reactive oxygen species generation by phagocytes encountered on the oral epithelium. Likewise, H7A79_RS09130, encoding a homolog of PglC showed different abundance patterns between gut and oral sites. In *N*. *gonorrhoeae* and *N*. *meningitidis*, pilin glycosylation, in which PglC plays a part, facilitates adherence of the pilus fiber to receptors on the epithelial cell surface [91]. That PglC is required for persistence in the oral cavity but not the gut may reflect a similar function for *N*. *musculi* pilin glycosylation, in which close interaction with the epithelial surface is modulated in part by post-translational modification of the Tfp fiber. Future work will provide a better understanding of the reasons for these site specificities.

A repertoire of pathogenic *Neisseria* genes, defined as modulating interactions of the bacteria with various host components, have been identified previously [24,48,52, 54–56,67,82,92]. Forty-five of these are identified in our *in vivo* screen, confirming their involvement in *Neisseria*-host relationships. As predicted by *in vitro* experimentation and our own work developing the *N*. *musculi*-mouse model system, Tfp mutants are underrepresented in oral and gut samples at weeks 6 and 8 post-inoculation, compared to the inoculum. Thirteen Tfp genes appear to be required for long-term colonization of at least one mucosal site. Mutants encoding Tfp structural proteins PilM, PilG, PilN, are underrepresented in both oral cavity and gut samples, as predicted by the model of Tfp core biogenesis [78]. Also underrepresented are mutants of *dacC*, which has been implicated in peptidoglycan remodeling during Tfp fiber extension [65].

Modification of the pilus appears equally important for niche maintenance. *pglB* and *pglC* mutants, which likely cannot glycosylate PilE, the major Tfp subunit, are unable to colonize the oral cavity and gut, although populations of *pglC* mutants rebounded in gut samples at weeks 6 and 8. Mutants of minor pilin genes *comP*, *pilH*,*I*,*J*, and *pilK* are also defective in long-term colonization of both host sites. Mutants of *pilZ*, which encodes a protein with a cyclic-di-GMP binding domain, were significantly depleted in gut samples only at week 8. This may indicate a previously undescribed role for *pilZ* in host interaction.

Extension and retraction of the Tfp fiber are the basis of *Neisseria* twitching motility, microcolony/biofilm formation, and activation of mechanosensitive host cell pathways [47,93–96]. These processes are carried out by 4 ATP-hydrolyzing motor proteins: PilF for fiber extension, and PilT, PilU and PilT2 for fiber retraction [71,97,98]. PilT, PilU and PilT2 are thought to work in concert to retract the pilus in an environment-dependent context, but to date little is known about their roles in persistent carriage [98,99]. *pilF* and *pilT2* mutants are underrepresented in oral cavity and gut samples, strongly suggesting that modulation of pilus movement is vital to host interactions. Notably, *pilF* has a more profound defect than *pilT2*. This may indicate a hierarchy of essentiality among these motor proteins: Tn5 insertion into *pilF* likely creates a non-piliated cell, ablating the dynamic action of Tfp entirely. Conversely, the apparent redundancy of the *pilT*, *pilU and pilT2* retraction motor genes suggests that multiple avenues exist for Tfp retraction. In the absence of one motor, the remaining motors may be able to compensate under specific host conditions. We were surprised by the absence of *pilE* (H7A79_RS10130) in our host essential candidate pool as prior work has demonstrated a requirement for the major pilin subunit in colonization [9]. However, in that study loss of *pilE* did not completely abrogate colonization in CAST/EiJ mice, which may explain our results. Further work is required to examine the interplay of major and minor pilins during host persistence.

The polysaccharide capsule is widely considered a primary determinant of bacterial pathogenicity. Many commensal bacteria are now known to produce a capsule, and this structure is important for survival in polymicrobial host environments [100–103]. Gene clusters encoding *cps* biosynthesis, transport, and translocation enzymes analogous to the *N*. *meningitidis* locus have been identified in 13 commensal *Neisseria* species, including *N*. *musculi* [88]. As the *N*. *meningitidis* cps provides resistance to antimicrobial peptides and evasion of opsonophagocytosis and complement, it likely provides a fitness advantage to the bacterium on mucosal surfaces regardless of the ability of the clinical isolate to invade the host [104–106]. This idea is supported by our findings. *N*. *musculi* *cps* Tn5 mutants are significantly underrepresented in gut and oral samples compared to the inoculum; this finding was validated by the defective behavior of an engineered *cps* promoter deletion mutant in long-term oral cavity colonization of A/J mice.

The structure and function of the *N*. *musculi* cps is not yet known. The divergence and rearrangement of *cps* biosynthesis and modification genes across the genus suggests that *Neisseria* species produce a wide range of capsule types, possibly with niche-specific functions. The *N*. *musculi* cps mutants identified in our screen are disrupted in capsule biosynthesis and transport (Regions A and C). While the transport locus is similar in other animal- and human-adapted *Neisseria*, we do not yet know what unique modifications may be caused by the uncharacterized genes in *N*. *musculi* Region A, or what impact these modifications may have on host interaction.

In *N*. *musculi*, the noncoding DNA segment between the divergently transcribed *cps* Region A and C contains several potential regulatory sites. The presence of putative Npa and Sigma54 recognition sequences strongly suggests that *N*. *musculi* capsule expression is dependent on environmental cues [107,108]. This idea is further supported by the presence of a 17 bp direct repeat upstream of the capsule transport region. In *N*. *meningitidis*, an 8 bp direct repeat in the 5’ UTR of *cssA* serves as a post-transcriptional thermo-controller [109,110]. It is tempting to theorize that a similar mechanism may regulate *cps* expression in *N*. *musculi* as well.

Among *Neisseria* species, *N*. *meningitidis* and *N*. *gonorrhoeae* have been shown to produce phage, and the function of these phages is not entirely defined [111–113]. Perhaps one of the most intriguing findings from our study is that phage related genes may influence *N*. *musculi* long-term carriage. Forty-seven genes identified by our screen are phage related; 29 of these cluster in a single region annotated as a prophage genome. Under-represented phage associated ORFs were identified from 3 intact putative prophage regions. Several of these genes may normally function in phage immunity, a vital defense in a phage-rich polymicrobial environment; however, many of these candidates have no annotated function. A substantial body of work has shown that phages play a role in genetic exchange between bacteria, including in *Neisseria* [87,111,112,114]. In these scenarios, phages may coincidentally transfer adaptation mechanisms and regulatory elements that have a fitness benefit for the new host in a wide range of environmental conditions. Studies have shown that temperate phage may increase the competitiveness of host bacteria in the niche by killing or lysogenizing unrelated bacteria lacking immunity, by the production of bacteriocins, and by mutualistic mechanisms that enhance biofilm formation [115,116]. The role these phages play in *Neisseria* long-term colonization remains to be elucidated.

Importantly, genes with unknown or predicted function comprise most of our mutant pool. Approximately 20% of candidate host interaction genes are annotated as encoding hypothetical proteins or proteins containing a domain of unknown function (DUF). A large proportion of these proteins display high similarity to those produced by human commensal and pathogenic *Neisseria*. Of 94 hypotheticals and 18 DUFs identified by our screen, 54 were highly similar to proteins produced by human adapted species, with half of these genes also encoded by *N*. *meningitidis* and/or *N*. *gonorrhoeae* (S5 Data). Despite the possibility of false positives due to the moderate saturation of our library, and potential bottlenecks presented by the screen itself, the prevalence of genes of unknown function in our candidate pool underlines how little we know of the processes needed by *Neisseria* to maintain a stable niche in its host.

The high degree of genetic similarity across the *Neisseria* genus suggests that commensals can offer key insights into how *N*. *meningitidis* and *N*. *gonorrhoeae* cause asymptomatic and persistent carriage. While certain *N*. *meningitidis* strains cause outbreaks of meningitis and septicemia, many others exist in the oropharynx of healthy adults and rarely if ever cause disease [21]. *N*. *gonorrhoeae* can also persist in women and men without eliciting symptoms [22]. As asymptomatic infection contributes to transmission, defining the commensal *Neisseria-*host relationship may reveal untapped strategies for preventing or treating *N*. *meningitidis* and *N*. *gonorrhoeae* infections. Novel host interaction factors common in this genus may represent new drug targets and potential vaccine antigens. Likewise, bacterial competition mechanisms and behaviors can be subverted or mimicked as treatment strategies.

While our screen was used to identify genes required for persistence, it could easily be adapted to examine the loci driving initial interactions, as well as the repertoire required by the bacterium to compete with the extant microbiota in the early phase of colonization. We note that our system has certain limitations: extra care must be taken to avoid over interpretation of results without validation of these mutants with clean deletions. While we attempted to address potential bottlenecks at the initial point of colonization through replicative experimentation, dose variation, and normalization and analysis of pooled samples from multiple mice, the possibility of false positives remains. Comparisons of library replicate gene insertion indices and read counts in both the inoculum and host passage samples shows a high degree of similarity by Pearson correlation analysis with r values ranging from 0.868–0.996 (S3 Table), suggesting that while a mild bottleneck may exist it may not drastically impact our results. Moreover, the moderate saturation of the library may mean that we have missed some genes that play a vital role in bacteria-host interactions, by assigning them to the housekeeping gene set. Despite these drawbacks, the results presented here still represent an important first step in identifying the repertoire of *Neisseria* genes essential for niche maintenance.

Finally, our finding that many *N*. *musculi* genes required for long-term colonization are homologs of *N*. *meningitidis* and *N*. *gonorrhoeae* virulence genes argues for a more nuanced definition of the term "virulence factor". We and others have in the past used "host-interaction factor" in its stead, and our findings here argue in favor of taking this approach. The conservation of these "virulence gene" homologs across multiple animal and human adapted *Neisseria* species, and their importance *in vivo*, suggest that differential regulation of gene expression during colonization and niche maintenance may influence infection outcome more than the presence or absence of a gene. Understanding the function of these genes during commensal carriage will give insight into *Neisseria* pathogenesis and shed light on the transition from commensal to pathogen and back.

## Supporting information

S1 Fig Housekeeping gene analysis of *N*. *musculi*. A. Bio-Tradis tradis_essentiality.R analysis of library passage 1 (top panel) and 2 (bottom panel) identified approximately 1100 genes with a lower than anticipated insertion index, suggesting a potential *in vitro* fitness defect. Insertion index thresholds were determined by log-odds ratios calculated on fits of gamma distributions of insertion indices in each sample. Histogram depicts the distribution of gene insertion indices from each replicate, and their calculated essentiality designation threshold. A gene insertion index of 0 indicates the gene may be required for growth, while an insertion index of 1 indicates full transposon saturation. Only genes below the calculated essential changepoint in both library passage samples were considered part of the housekeeping gene set, after repetitive elements were removed from further analysis. This resulted in 968 genes designated as putative housekeeping candidates with a potential fitness defect *in vitro*. 192 genes had no insertions, while 776 had fewer insertions than expected. Candidates in this gene set were excluded from analysis of host passage samples to prevent false positives due to *in vitro* fitness defects and/or low initial saturation. B-C. Pathway identification of housekeeping candidates. Candidate genes appearing in both library passage samples were analyzed using the clusterProfiler enrichKEGG tool using the *N*. *musculi* AP2031 KEGG annotation. B. Barplot provides an overview of genes mapping to a subset of 20 pathways. Bar color corresponds to Benjamin-Hochberg adjusted p-value of clusterProfiler gene enrichment results. Components of the ribosome and bacterial secretion systems were significantly over-represented in the housekeeping candidate pool. The remaining genes mapped to pathways associated with other central physiologic processes, including oxidative phosphorylation, lipid metabolism, and homologous recombination and repair, although these pathways were not significantly enriched. C. Gene concept network plot depicts pathway association from (B) at the gene level for a subset of putative housekeeping genes. Nodes (tan dots) represent pathways labeled by edge color, while dot size corresponds to the number of candidate genes mapping to that pathway. Genes are labeled with their Genbank locus tag corresponding to the KEGG annotation.
(PDF)

S2 Fig Pathway enrichment analysis of host interaction candidate genes. To identify pathways involved in host niche maintenance, 325 candidate genes with significantly altered abundance in all host passage samples were analyzed using the clusterProfiler enrichKEGG tool and the *N*. *musculi* AP2031 KEGG annotation. A. Bar plot depicts pathways whose genes are overrepresented in host samples. Bar color corresponds to Benjamin-Hochberg (BH) adjusted p-value of clusterProfiler enrichKegg over-representation analysis results. A BH adjusted *p <* 0.1 was considered significant. Metabolic processes, including biosynthesis of amino acids and secondary metabolites, and chemotaxis, were significantly over-represented, suggesting these pathways are important for host adaptation. B. Gene network plot depicts clustering of host enriched pathways to identify functionally related processes. Nodes represent pathways identified in (A) and node size indicates gene count. Node color indicates BH adjusted *p* value from pathway overrepresentation results. Edges indicate genes shared between pathways. Similarity between nodes was calculated using Jaccard’s similarity coefficient method in clusterProfiler. Cluster formation around amino acid degradation, gluconeogenesis and the TCA cycle suggests interconnection between these pathways from a highly overlapping gene set.
(TIF)

S3 Fig The capsule locus of *N*. *musculi* is regulated by the *ctr*A-A1/*csi*A intergenic region. cDNA was generated from Wt *N*. *musculi* total RNA, and primers flanking 5’ and 3’ ends of consecutive genes were used to amplify transcripts transcribed from the 228 bp intergenic region. While multi-gene transcripts (e.g., A5-A7) were not reliably generated, PCR products were routinely generated using primers spanning junctions of adjacent genes in the locus *ctr*A-D (Region C) and A1-*ctr*F (Region A, B), indicating these genes are co-transcribed. Top panel: Wt genomic DNA (gDNA) control; middle panel: RT-PCR of wt cDNA; bottom panel: -RT controls. M, 1 kb plus DNA marker.
(TIF)

S4 Fig Deletion of the capsule locus intergenic sequence does not affect growth rate. Bacteria were suspended in supplemented GC broth to an initial OD_600_ = 0.05 and optical density was measured for 8 hours. No significant differences in growth was observed between Wt, Δ*cps*228 mutants and the complemented strain over this time.
(TIF)

S5 Fig Comparison of COG frequency between ORFs unique to gut and oral samples. 48 host interaction candidate genes were unique to oral samples while 63 were unique to the gut. However, no significant difference in COG frequency of annotated ORFs was observed between Oral and Gut host interaction candidates by Two-sided Fisher’s exact test. Category definitions based on the Database of Clusters of Orthologous Genes as follows: J, Translation, Ribosomal Structure and Biogenesis; K, Transcription; L, Replication, Recombination and Repair; B, Chromatin Structure and Dynamics; D, Cell cycle control, cell division and chromosome partitioning; V, Defense Mechanisms; T, Signal Transduction Mechanisms; M, Cell Wall/Membrane/Envelope Biogenesis; N, Cell Motility; W, Extracellular Structures; U, Intracellular Trafficking, Secretion, and Vesicular Transport; O, Posttranslational Modification, Protein Turnover, Chaperones; C, Energy Production and Conversion; G, Carbohydrate Transport and Metabolism; E, Amino Acid Transport and Metabolism; F, Nucleotide Transport and Metabolism; H, Coenzyme Transport and Metabolism; I, Lipid Transport and Metabolism; P, Inorganic Ion Transport and Metabolism; Q, Secondary Metabolite Biosynthesis, Transport and Catabolism.
(TIF)

S1 Table Primers, synthetic sequences, and strains utilized in this study. (PDF)

S2 Table A selection of genes identified as candidate host interaction factors. Genes are grouped by putative pathway association identified by KEGG functional annotation. Amino acid sequence comparisons were computed from KEGG Ortholog Gene Function Identification (GFIT) Sequence similarity database tables using the *N*. *musculi* Genbank ID. ^1^Sequence analyzed by BLASTP to identify any similarity with human adapted *Neisseria*. Query coverage is reported for BLASTP comparisons. Species abbreviations are shown according to KEGG nomenclature for strain designation. Nme, *Neisseria meningitidis* MC58; Nm*, *Neisseria meningitidis* serogroup I/93004; Nel, *Neisseria elongata* sups. *glycolitica* ATCC29315; Ngo, *Neisseria gonorrhoeae* FA1090; Nla, *Neisseria lactamica* 02–06; Nsi, *Neisseria mucosa* FDAARGOS; Nfv, *Neisseria flavescens* ATCC 13120; Ncz, *Neisseria cinerea* NCTC10294; Nci, *Neisseria canis* NCTC10296; Nbc, *Neisseria baciliformis* DSM 233383; Nmj, *Neisseria mucosa* ATCC 19696; Nani, *Neisseria animaloris* NCTC12227; Nsf, *Neisseria subflava* ATCC 49275; Nzo, *Neisseria zoodegmatis* NCTC12230; Nsg, *Neisseria shayeganii* DSM22244; Nsc, *Neisseria sicca* NS20201025; Nwd, *Neisseria wadsworthii* DSM22245; Nwe, *Neisseria weaver* NCTC13585.
(PDF)

S3 Table Correlation of read counts and insertion indexes by replicate. Comparisons between library replicates 1 and 2 in each sample type, and between sample types in each replicate, were performed by Pearson r correlation analysis in GraphPad Prism V9.
(PDF)

S1 Data Candidates with predicted *in vitro* fitness defects and/or lower than expected gene insertion indices by tradis_essentiality testing. Calculations for determining a fitness defect were made by tradis_essentiality.R analysis of library 1 and 2 passage samples. Repetitive transposable elements were removed from this data set, as multi-mapped reads were ignored during the insertion quantification step, giving these open reading frames an insertion index of 0. Gene insertion index distribution changepoints were calculated at 0.0149 for passage 1 and 0.0087 for passage 2 samples. Only genes failing to pass the calculated thresholds in both samples were included in the putative housekeeping gene pool. A description of analysis methods can be found in the Materials and Methods section. Pathway and COG prediction was performed by eggNOGmapper-V2 analysis and comparisons to the KEGG database using the *N*. *musculi* reference genome. Transposon insertions for each ORF are listed for each sample output. Gene insertion indices (Tn5 insertions normalized to gene length) are listed for passage 1 and 2 samples. Insertion counts are listed for all samples.
(XLSX)

S2 Data Comparison of Tn5 directed reads from library replicate 1 and 2 host samples to inoculum. Tn5 directed reads from library replicate 1 and 2 host samples were compared to the inoculum by EdgeR analysis implemented in the Bio-Tradis pipeline. Genes listed met significance criteria (logFC < -1, > 1, q < 0.05) in; A. all three host samples; B., oral and week 6 gut samples; C., oral and week 8 gut samples; D., week 6 and week 8 gut samples; E., only oral samples; F., week 8 gut samples alone; and G., week 6 gut samples alone. Candidates with a potential *in vitro* fitness defect (those ORFs with lower than anticipated gene insertion indices by tradis_essentiality.R analysis) were removed to reduce potential false positives. KEGG pathway and COG category was predicted by analysis of the *N*.*musculi* Genbank annotation using eggNOG Mapper-v2.
(XLSX)

S3 Data *Neisseria* host interaction homologs meeting significance criteria in analysis of mouse passage samples. A. Host interaction candidates with high similarity to *N*. *gonorrhoeae* and *N*. *meningitidis* virulence genes. Genes were identified based on analysis of host-microbe interaction genes listed in B. *N*. *gonorrhoeae* and *N meningitidis* comparisons are listed for MC58 and FA1090. H7A79_1831 comparison is based on blastp comparison to CCP19763 from *N*. *meningitidis* serogroup K strain ST-8742, BIGSDB alt. ID NEIS2179. Fold changes in red did not meet fold change or q value significance criteria. B. Identification of *N*. *musculi* genes sharing high similarity to loci associated with host interaction in *N*. *gonorrhoeae* and *N*. *meningitidis*. Comparisons shown are pulled from KEGG Sequence Similarity Database (SSDB) Gene Function Identification tool (GFIT) tables, computed by Smith-Waterman similarity search. If high similarity to *N*. *gonorrhoeae* or *N*. *meningitidis* for a particular locus was not identified, data is displayed for the top hit from any human adapted *Neisseria* species.
(XLSX)

S4 Data Summary of phage related genes identified in host interaction candidate pool. A. Host interaction candidate genes found within putative prophage regions identified in PHASTER analysis. Similar *N*. *meningitidis* and *N*. *gonorrhoeae* genes reported from KEGG database GFIT SSDB Tables or (*) blastp Searches. Genes are listed with Tn5 directed read fold change from BioTradis analysis. B. Overview of PHASTER prophage analysis performed on *N*. *musculi* reference sequence. 11 regions were identified as putatively intact prophages or incomplete regions based on comparison to known phage genomes. C. Per Region breakdown of *N*. *musculi* ORFs. Log_2_FC of significant hits in host passage samples is listed when applicable. * Fold change and/or Refseq ID in red text indicates gene was identified in putative fitness defect set and was ignored in final analysis of host interaction candidates. ns = not significant. D. Identification of *N*. *musculi* genes with similarity to *Neisseria* filamentous and Mu-type prophages. *N*. *gonorrhoeae* and *N meningitidis* ORFs encoded in prophage regions were searched against the *N*. *musculi* genome using KEGG GFIT SSDB searches and blastp.
(XLSX)

S5 Data Host interaction candidate genes with unknown function. Genes annotated as hypothetical (A) or encoding a domain of unknown function (B) meeting significance criteria in at least one host site were analyzed to identify similarities with other *Neisseria* species. ORFs are listed with results of tradis.comparison.R analysis. Genes with similarity to those encoded by either human commensal, pathogen, or animal commensal species are color coded according to similarity key. Percent identity is reported from KEGG database GFIT SSDB searches by *N*. *musculi* Genbank locus tag. Species abbreviations are shown according to KEGG nomenclature for strain designation. Nme, *Neisseria meningitidis* MC58; Nel, *Neisseria elongata* sups. *glycolitica* ATCC29315; Ngo, *Neisseria gonorrhoeae* FA1090; Nla, *Neisseria lactamica* 02–06; Nsi, *Neisseria mucosa* FDAARGOS; Nfv, *Neisseria flavescens* ATCC 13120; Ncz, *Neisseria cinerea* NCTC10294; Nci, *Neisseria canis* NCTC10296; Nbc, *Neisseria baciliformis* DSM 233383; Nmj, *Neisseria mucosa* ATCC 19696; Nani, *Neisseria animaloris* NCTC12227; Nsf, *Neisseria subflava* ATCC 49275; Nzo, *Neisseria zoodegmatis* NCTC12230; Nsg, *Neisseria shayeganii* DSM22244; Nsc, *Neisseria sicca* NS20201025; Nwd, *Neisseria wadsworthii* DSM22245; Nwe, *Neisseria weaveri* NCTC13585.
(XLSX)
