# Supplementary figures and images for: Neisseria genes required for persistence identified via in vivo screening of a transposon mutant library
Source: PLoS Pathog. 2022 May 17;18(5):e1010497. doi: 10.1371/journal.ppat.1010497 (PMC9140248; doi:10.1371/journal.ppat.1010497)

S1 Fig A.

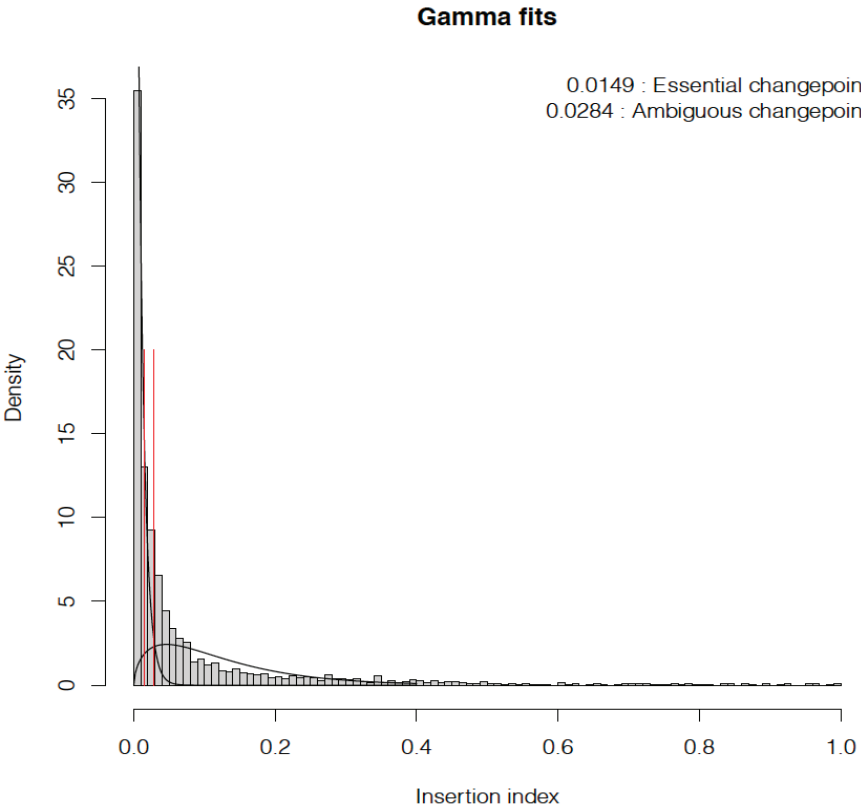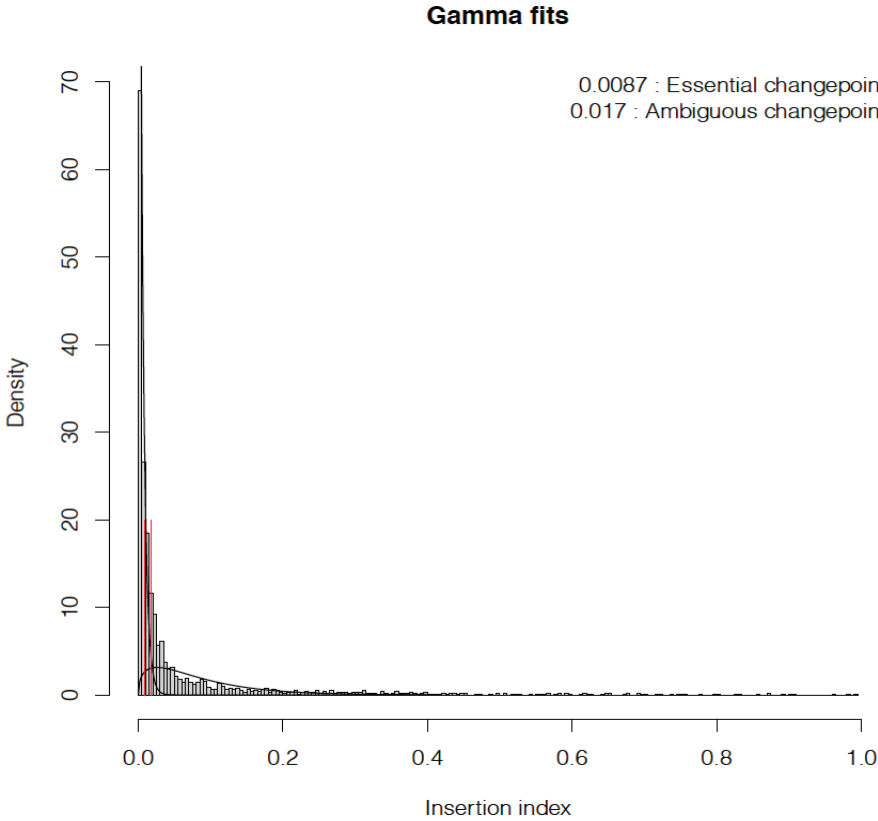

**B.**

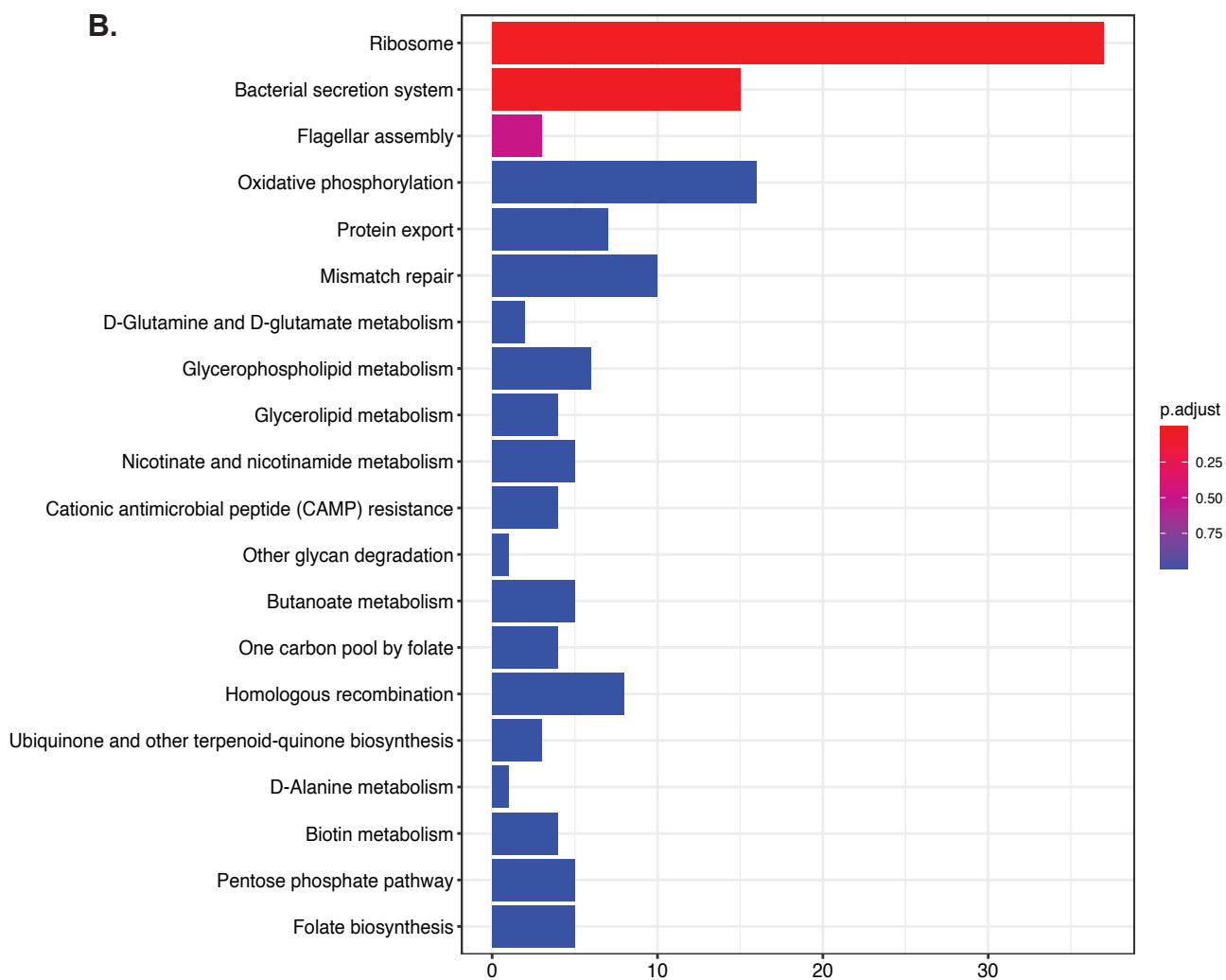

C.

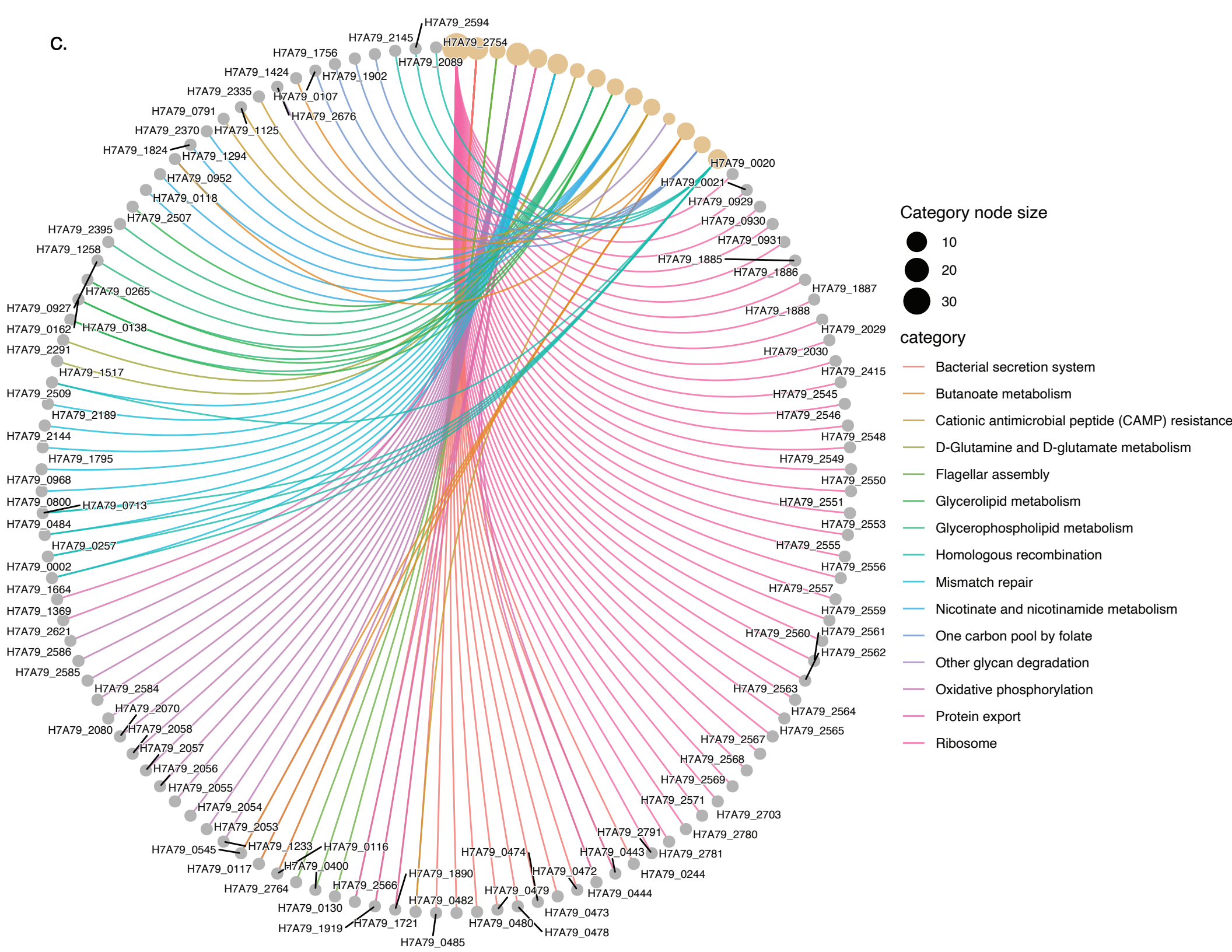

Supplement: S1 Fig — A. Bio-Tradis tradis_essentiality.R analysis of library passage 1 (top panel) and 2 (bottom panel) identified approximately 1100 genes with a lower than anticipated insertion index, suggesting a potential in vitro fitness defect. Insertion index thresholds were determined by log-odds ratios calculated on fits of gamma distributions of insertion indices in each sample. Histogram depicts the distribution of gene insertion indices from each replicate, and their calculated essentiality designation threshold. A gene insertion index of 0 indicates the gene may be required for growth, while an insertion index of 1 indicates full transposon saturation. Only genes below the calculated essential changepoint in both library passage samples were considered part of the housekeeping gene set, after repetitive elements were removed from further analysis. This resulted in 968 genes designated as putative housekeeping candidates with a potential fitness defect in vitro. 192 genes had no insertions, while 776 had fewer insertions than expected. Candidates in this gene set were excluded from analysis of host passage samples to prevent false positives due to in vitro fitness defects and/or low initial saturation. B-C. Pathway identification of housekeeping candidates. Candidate genes appearing in both library passage samples were analyzed using the clusterProfiler enrichKEGG tool using the N. musculi AP2031 KEGG annotation. B. Barplot provides an overview of genes mapping to a subset of 20 pathways. Bar color corresponds to Benjamin-Hochberg adjusted p-value of clusterProfiler gene enrichment results. Components of the ribosome and bacterial secretion systems were significantly over-represented in the housekeeping candidate pool. The remaining genes mapped to pathways associated with other central physiologic processes, including oxidative phosphorylation, lipid metabolism, and homologous recombination and repair, although these pathways were not significantly enriched. C. Gene concept [file ppat.1010497.s001.pdf]

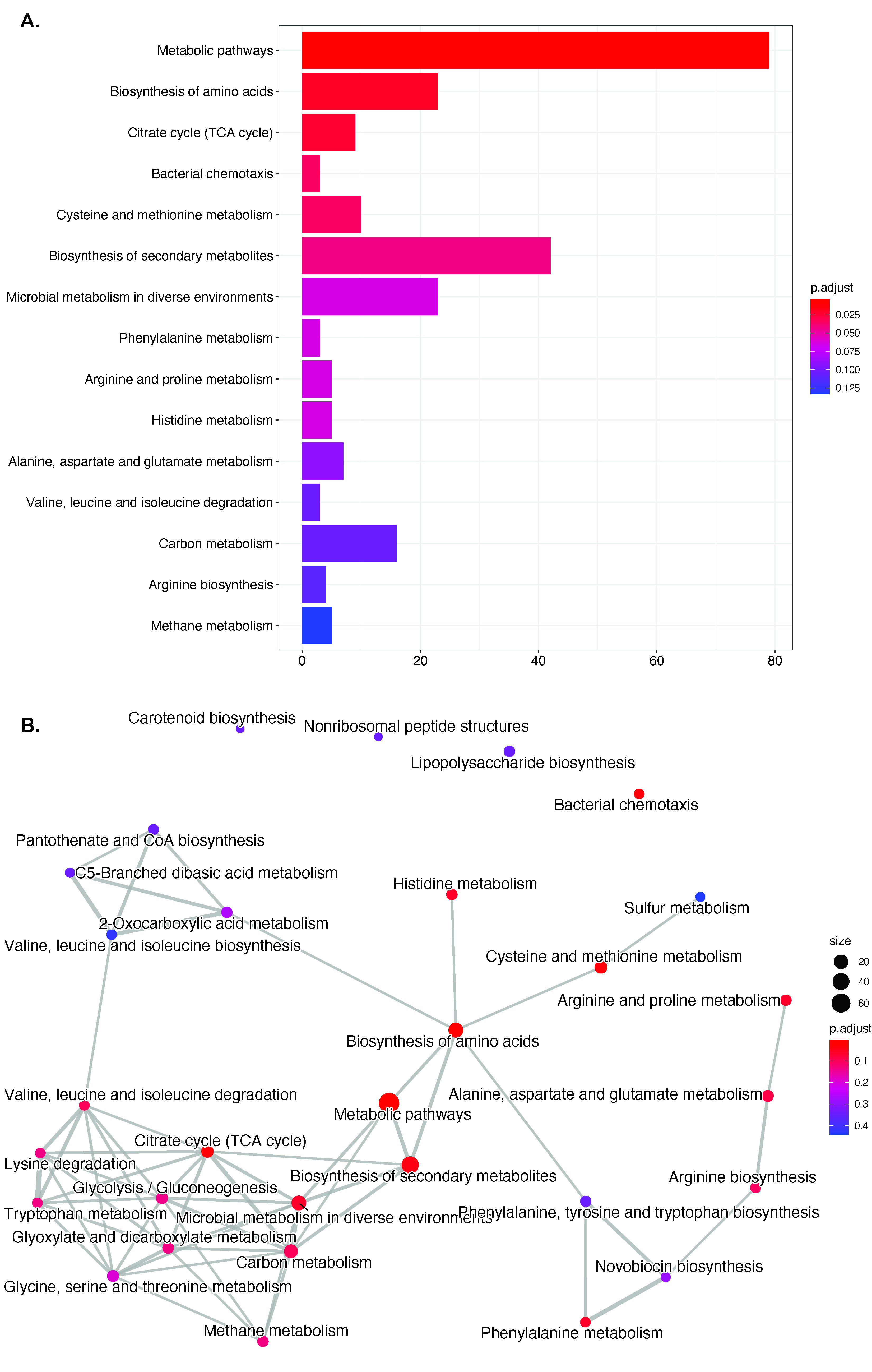

Supplement: S2 Fig — To identify pathways involved in host niche maintenance, 325 candidate genes with significantly altered abundance in all host passage samples were analyzed using the clusterProfiler enrichKEGG tool and the N. musculi AP2031 KEGG annotation. A. Bar plot depicts pathways whose genes are overrepresented in host samples. Bar color corresponds to Benjamin-Hochberg (BH) adjusted p-value of clusterProfiler enrichKegg over-representation analysis results. A BH adjusted p < 0.1 was considered significant. Metabolic processes, including biosynthesis of amino acids and secondary metabolites, and chemotaxis, were significantly over-represented, suggesting these pathways are important for host adaptation. B. Gene network plot depicts clustering of host enriched pathways to identify functionally related processes. Nodes represent pathways identified in (A) and node size indicates gene count. Node color indicates BH adjusted p value from pathway overrepresentation results. Edges indicate genes shared between pathways. Similarity between nodes was calculated using Jaccard’s similarity coefficient method in clusterProfiler. Cluster formation around amino acid degradation, gluconeogenesis and the TCA cycle suggests interconnection between these pathways from a highly overlapping gene set. (TIF) [file ppat.1010497.s002.tif]

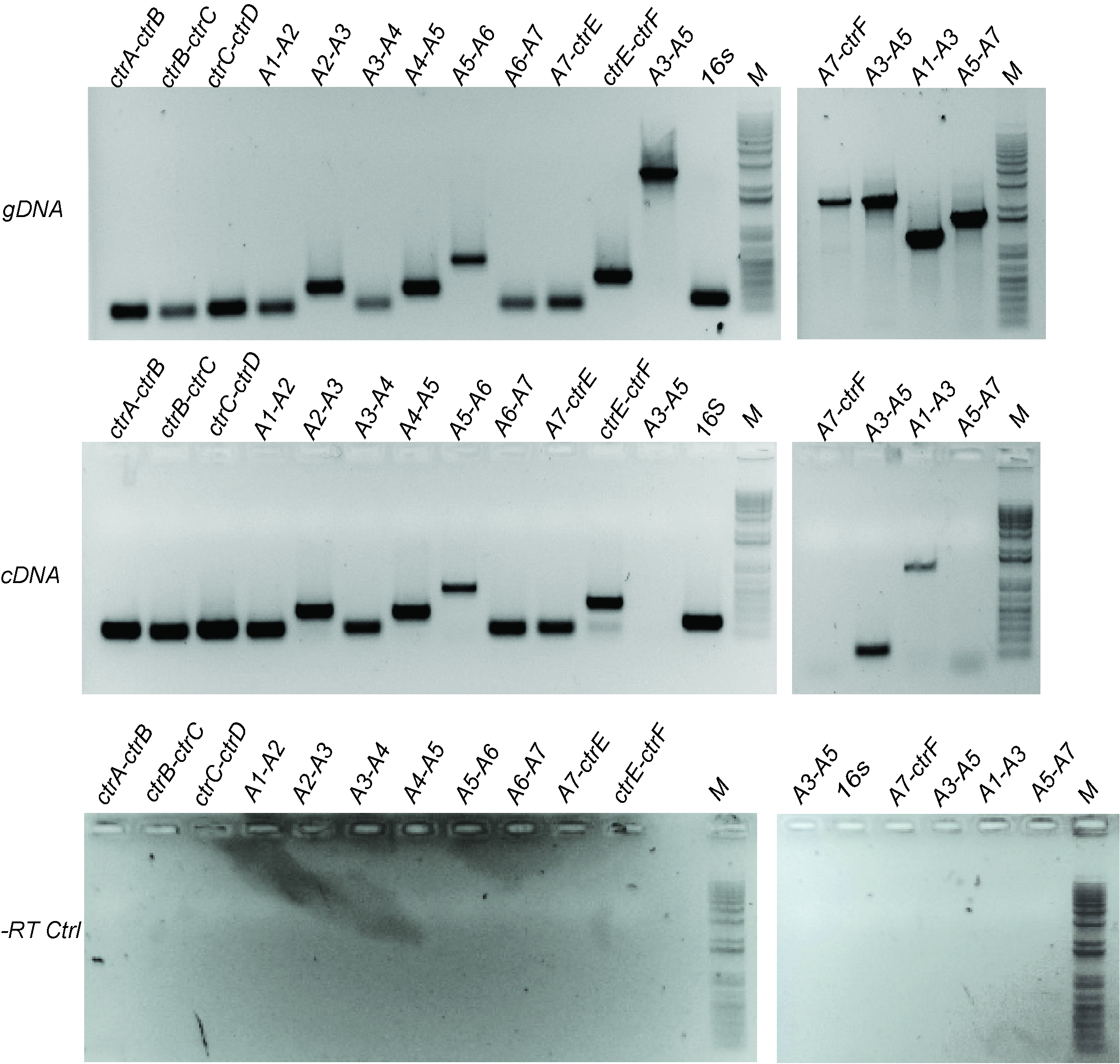

Supplement: S3 Fig — cDNA was generated from Wt N. musculi total RNA, and primers flanking 5’ and 3’ ends of consecutive genes were used to amplify transcripts transcribed from the 228 bp intergenic region. While multi-gene transcripts (e.g., A5-A7) were not reliably generated, PCR products were routinely generated using primers spanning junctions of adjacent genes in the locus ctrA-D (Region C) and A1-ctrF (Region A, B), indicating these genes are co-transcribed. Top panel: Wt genomic DNA (gDNA) control; middle panel: RT-PCR of wt cDNA; bottom panel: -RT controls. M, 1 kb plus DNA marker. (TIF) [file ppat.1010497.s003.tif]

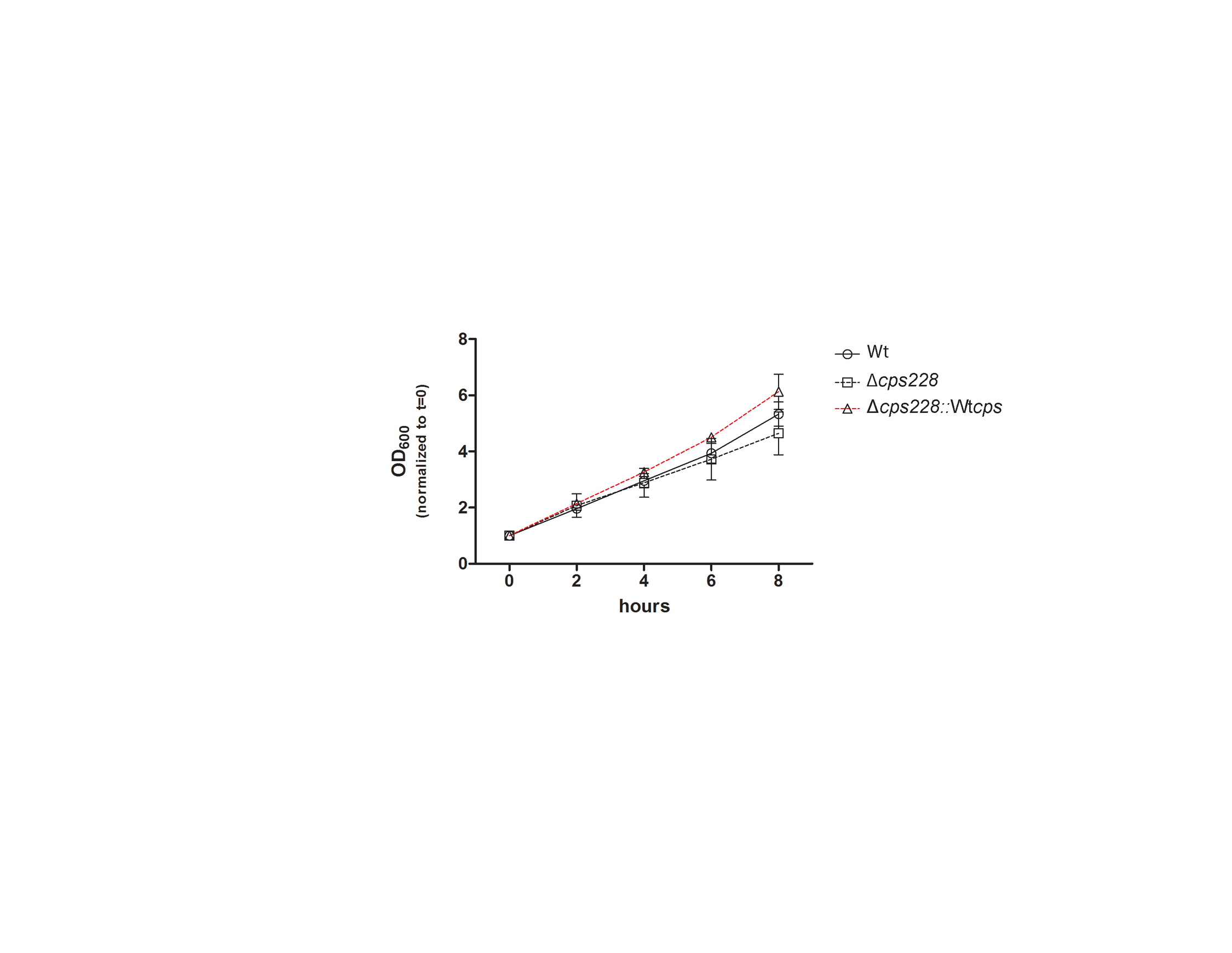

Supplement: S4 Fig — Bacteria were suspended in supplemented GC broth to an initial OD600 = 0.05 and optical density was measured for 8 hours. No significant differences in growth was observed between Wt, Δcps228 mutants and the complemented strain over this time. (TIF) [file ppat.1010497.s004.tif]

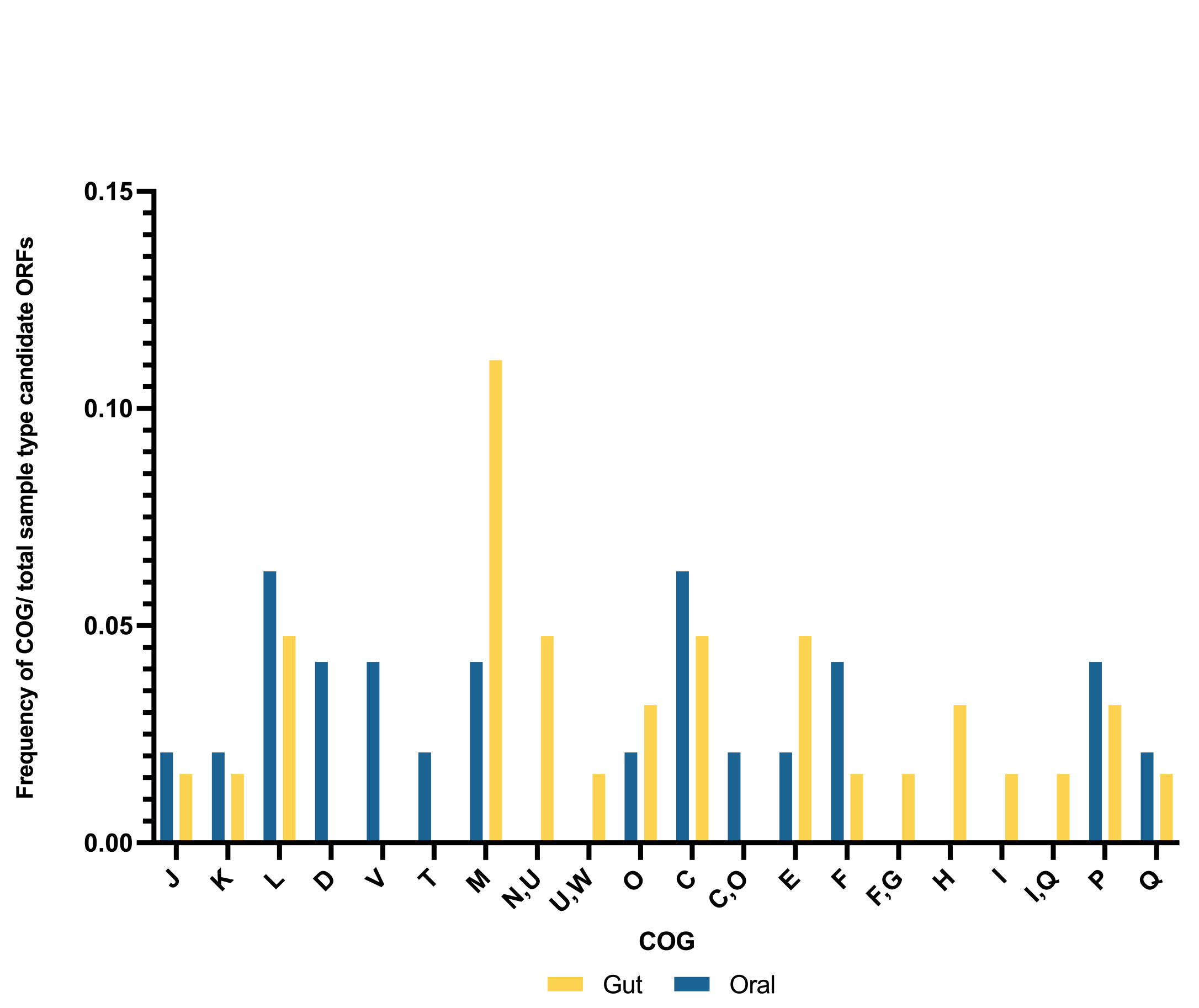

Supplement: S5 Fig — 48 host interaction candidate genes were unique to oral samples while 63 were unique to the gut. However, no significant difference in COG frequency of annotated ORFs was observed between Oral and Gut host interaction candidates by Two-sided Fisher’s exact test. Category definitions based on the Database of Clusters of Orthologous Genes as follows: J, Translation, Ribosomal Structure and Biogenesis; K, Transcription; L, Replication, Recombination and Repair; B, Chromatin Structure and Dynamics; D, Cell cycle control, cell division and chromosome partitioning; V, Defense Mechanisms; T, Signal Transduction Mechanisms; M, Cell Wall/Membrane/Envelope Biogenesis; N, Cell Motility; W, Extracellular Structures; U, Intracellular Trafficking, Secretion, and Vesicular Transport; O, Posttranslational Modification, Protein Turnover, Chaperones; C, Energy Production and Conversion; G, Carbohydrate Transport and Metabolism; E, Amino Acid Transport and Metabolism; F, Nucleotide Transport and Metabolism; H, Coenzyme Transport and Metabolism; I, Lipid Transport and Metabolism; P, Inorganic Ion Transport and Metabolism; Q, Secondary Metabolite Biosynthesis, Transport and Catabolism. (TIF) [file ppat.1010497.s005.tif]
