# Supplementary material for: Neisseria genes required for persistence identified via in vivo screening of a transposon mutant library
Source: PLoS Pathog. 2022 May 17;18(5):e1010497. doi: 10.1371/journal.ppat.1010497 (PMC9140248; doi:10.1371/journal.ppat.1010497)
Supplement: S1 Table — (PDF) [file ppat.1010497.s006.pdf]

S1 Table: Primers, Synthetic Sequences and Strains utilized in this study

Strain	Species	Notes	Source
AP2365	*N. musculi*	Rif resistant derivative of AP2031 type strain	(1-3)
AP2365 ∆cps228	*N. musculi*	Km resistant capsule null derivative	This study
AP2365∆cps228 ::wt cps	*N. musculi*		This study
MS11	*N. gonorrhoeae*		(4)
FAM18	*N. meningitidis*		(5)

Primer	Use	Sequence	Source
Ind_Ad_T	Library preparation	ACACTCTTTCCCTACACGACGCTCTTCCGATC*T	(6)
Ind_Ad_B	Library preparation	GATCGGAAGAGCGGTTCAGCAGGAATGCCGAGACCGATCTC	(6)
Tn-FO	Tn5 Insertion site enrichment PCR	TCGTCGGCAGCGTCAGATGTGTATAAGAGACAGCGGGGATCCTCTAGAGTCGACCTGC	(6)
Adapt-RO		GTCTCGTGGGCTCGGAGATGTGTATAAGAGACAGACACTCTTTCCCTACACGACGCTCTTCCGATC	(6)
MR493	Detect *16*S transcript	ATCCTGGCTCAGATTGAACG	(1)
MR494		CCGCTTTCCTTCTCAAAGTG	
IM017	Detect *ctr*A transcript	AGTATCCGTATGCCGCTCAC	(1)
IM018		AGCTGCACCGAAATATCCTG	
IM086	Detect *ctr*B transcript	TTAAAGAAGCGGCAGGAGAG	This study
IM087		ACTTGGTCAAGCTGGGTTTG	
IM088	Detect *ctr*C transcript	ATGCGGTAGGAAAACTGTGG	This study
IM089		ACCCACAAAGCGTATTCCTG	
IM090	Detect *ctr*D transcript	CCGACTAAAGGCGAGATCAG	This study
IM091		AAAGCGCAAATTATCCATGC	
IM019	Detect *A1* transcript	CAGATGGACGTTGTGTTTGC	(1)
IM020		AGGGAAAATTTCGGAGAAGG	
IM094	Detect *A2* transcript	ATTGATCTCACTGGCCAACC	This study
IM095		GGTCGGGTGTTTTATTCACG	
IM096	Detect *A3* transcript	ACAGCGTAACCAGACCTTGG	This study
IM097		AGCCGGGTCTTCTTTATTCC	
IM098	Detect A4 transcript	TCCTGCCAATCGGATTTTAG	This study
IM099		TTTAATCAGGCTGGGTACGG	
IM100	Detect A5 transcript	TGGGGCAGTAGCTTTCTTTC	This study
IM101		AGGGCAGCAATACCTTTTCC	
IM102	Detect *A6* transcript	CGACCATAAATGGGGTGTTC	This study
IM103		ATGAACAGCGGCAGGTAATC	
IM104	Detect A7 transcript	TTCGACCGCTTTATCGATTC	This study
IM105		ACGGGAAACATCGCTACATC	
IM021	Detect *ctr*E transcript	CATAAAAAGGCAGGCCGTAG	(1)
IM022		GACCAAACCGTAACCGAATG	
IM023	Detect *ctr*F transcript	AGCTGCCTTGAAAGGTGATG	(1)
IM024		GTTACGCTCAACAGCACCAG	
IM110		CGCCGATTGTCAGTAGTGTG	

IM111	Detect *ctr*A-*ctr*B transcript	TAGCAGCTGTTGCCTGTTTC	This study
IM112	Detect *ctr*B-*ctr*C transcript	ATCGGTTTGATGCTTTACGG	This study
IM113		TTACCCGCCCTTGAATAACC	
IM114	Detect *ctr*C-*ctr*D transcript	CAAGTTCAGCAAAGGGGTTG	This study
IM115		AGAATGCCGATTTTCTCACC	
IM116	Detect *A1-A2* transcript	TACGCCGATAACTTTTACGC	This study
IM117		TGCCATTTAACGAGGAAAGC	
IM118	Detect *A2-A3* transcript	TTCTCAAGCTGTTCATCATCG	This study
IM119		TGTAGATTCCGTGGTGGTTG	
IM120	Detect *A3*-A4 transcript	CTTTAGGGAGGCAATACATGC	This study
IM121		TCGGCCAAACAATGGATTAC	
IM122	Detect A4-A5 transcript	CTGAAAACAGATGGGCAATG	This study
IM123		AACGGTCAGCGGACAAAG	
IM124	Detect A5-A6 transcript	GTTAAGGCATGGGCAGTTTG	This study
IM125		ACCGCCAATGGAACATTATC	
IM126	Detect A6-A7 transcript	GGAGGGAAAACCAACTGCTG	This study
IM127		TCTTAGAAGCAGAGATCAACAAGG	
IM128	Detect A7-*ctr*E transcript	AAGCCCGAAACTTGAGAAAAG	This study
IM129		GATTTGGAAATGGCCAAGG	
IM130	Detect *ctr*E-*ctr*F transcript	TCCGGGAATAAAGGTGTTTG	This study
IM131		AAACCGCCGTCAATATTCTG	
IM132	Detect *A1-A3* transcript	GATTCCGGCGGTATTCAAG	This study
IM133		TCCTGTTGGAAATAACGTTTCA	
IM134	Detect *A3*-A5 transcript	TGACGGAAATCGTATTGCTTT	This study
IM135		ACTCATTTCGGCCAAACAAT	
IM136	Detect *A3*-A5 (1) transcript	CTGAAAACAGATGGGCAATG	This study
IM137		GCACAAAAATTCACCGAAGC	
IM138	Detect A5-A7 transcript	CGGCGGGTATGCAGAATC	This study
IM139		ACAAACCGGCACAGCTTACT	
IM140	Detect A7-*ctr*F transcript	AGCCATTGCGAGAAGGTTTT	This study
IM141		AAGCGGCAGCTTTATTTGAA	
IM059	Detect Δ*cps*227::Kan mutant	TGCCGAATACAATCAGCAGT	This study
IM060		TCCACATCAACCACAGCAAC	This Study

IM132	Geneblock for Deletion of the 228bp IGR	**TTTGATAGCCTCCGGCCGGGTGCCGAATACAATCAGC<br>AGTTTTTTAGCACGTGCGGGGGGAGGGGATACCGCTA<br>TGGGATTAAAAGCAAATGAATCGTTGAATGTTGTCAAT<br>GTCAGAGCACTATTATCGGCGTTATTGTTTTTTTGCACG<br>GCACAGCCTTTCGTATTACACGGGTAGAACTTTGTCAT**<br>TTAAGGGATGCAGTTTATGCATCCCTTAACTCGAGGGC<br>TTGACACTTTATGCTTCCGGCTCGTATAATGTGTGGATA<br>GTGGGAGGAAAGCATGATTGAACAAGATGGATTGCACG<br>CAGGTTCTCCGGCCGCTTGGGTGGAGAGGCTATTCGG<br>CTATGACTGGGCACAACAGACAATCGGCTGCTCTGATG<br>CCGCCGTGTTCCGGCTGTCAGCGCAGGGGCGCCCGGT<br>TCTTTTTGTCAAGACCGACCTGTCCGGTGCCCTGAATG<br>AACTGCAGGACGAGGCAGCGCGGCTATCGTGGCTGGC<br>CACGACGGGCGTTCCTTGCGCAGCTGTGCTCGACGTT<br>GTCACTGAAGCGGGAAGGGACTGGCTGCTATTGGGCG<br>AAGTGCCGGGGCAGGATCTCCTGTCATCCCACCTTGCT<br>CCTGCCGAGAAAGTATCCATCATGGCTGATGCAATGCG<br>GCGGCTGCATACGCTTGATCCGGCTACCTGCCCATTCG<br>ACCACCAAGCGAAACATCGCATCGAGCGAGCACGTACT<br>CGGATGGAAGCCGGTCTTGTCGATCAGGATGATCTGGA<br>CGAAGAGCATCAGGGGCTCGCGCCAGCCGAACTGTTC<br>GCCAGGCTCAAGGCGCGCATGCCCGACGGCGAGGATC<br>TCGTCGTGACCCATGGCGATGCCTGCTTGCCGAATATC<br>ATGGTGGAAAATGGCCGCTTTTCTGGATTCATCGACTG<br>TGGCCGGCTGGGTGTGGCGGACCGCTATCAGGACATA<br>GCGTTGGCTACCCGTGATATTGCTGAAGAGCTTGGCGG<br>CGAATGGGCTGACCGCTTCCTCGTGCTTTACGGTATCG<br>CCGCTCCCGATTCGCAGCGCATCGCCTTCTATCGCCTT<br>CTTGACGAGTTCTTCTGAGCCGTCTGAAGTTTAAACATC<br>GATAAAATGCCGTCCGAAGCTTGAGTTCAGACGGCATT<br>TT**ATGACGCGTTTCCAGTTGCCGGCCGGATGCATTCTA<br>TTGCTGCTTTTAGGGTGTGGTGCTTTGCCTACTTCAGG<br>GCCGAATCAAACCAAGGTGATAAGTTTGCAAAATCAA<br>AGCCGCCAGGCGGCAGTGCCTGATGTTGCTGTGGTTG<br>ATGTGGACGAGCGGGTAATTTCCACACTGTAT**	This study
IM133	Capsule complementation	ACGCCTTCACGAAACAGATT	This study
IM134		AATTATTCTGCGCCACACG	

1. Ma M, Powell DA, Weyand NJ, Rhodes KA, Rendon MA, Frelinger JA, et al. A Natural Mouse Model for Neisseria Colonization. Infect Immun. 2018;86(5).
2. Rhodes K, Ma M, So M. A Natural Mouse Model for Neisseria Persistent Colonization. Methods Mol Biol. 2019;1997:403-12.
3. Weyand NJ, Ma M, Phifer-Rixey M, Taku NA, Rendon MA, Hockenberry AM, et al. Isolation and characterization of Neisseria musculi sp. nov., from the wild house mouse. Int J Syst Evol Microbiol. 2016;66(9):3585-93.

4. Edwards M, McDade RL, Schoolnik G, Rothbard JB, Gotschlich EC. Antigenic analysis of gonococcal pili using monoclonal antibodies. J Exp Med. 1984;160(6):1782-91.
5. Kawula TH, Aho EL, Barritt DS, Klapper DG, Cannon JG. Reversible phase variation of expression of Neisseria meningitidis class 5 outer membrane proteins and their relationship to gonococcal proteins II. Infect Immun. 1988;56(2):380-6.
6. McCarthy AJ, Stabler RA, Taylor PW. Genome-Wide Identification by Transposon Insertion Sequencing of Escherichia coli K1 Genes Essential for In Vitro Growth, Gastrointestinal Colonizing Capacity, and Survival in Serum. J Bacteriol. 2018;200(7).
