# Supplementary material for: Neisseria genes required for persistence identified via in vivo screening of a transposon mutant library
Source: PLoS Pathog. 2022 May 17;18(5):e1010497. doi: 10.1371/journal.ppat.1010497 (PMC9140248; doi:10.1371/journal.ppat.1010497)
Supplement: S2 Table — Genes are grouped by putative pathway association identified by KEGG functional annotation. Amino acid sequence comparisons were computed from KEGG Ortholog Gene Function Identification (GFIT) Sequence similarity database tables using the N. musculi Genbank ID. 1Sequence analyzed by BLASTP to identify any similarity with human adapted Neisseria. Query coverage is reported for BLASTP comparisons. Species abbreviations are shown according to KEGG nomenclature for strain designation. Nme, Neisseria meningitidis MC58; Nm*, Neisseria meningitidis serogroup I/93004; Nel, Neisseria elongata sups. glycolitica ATCC29315; Ngo, Neisseria gonorrhoeae FA1090; Nla, Neisseria lactamica 02–06; Nsi, Neisseria mucosa FDAARGOS; Nfv, Neisseria flavescens ATCC 13120; Ncz, Neisseria cinerea NCTC10294; Nci, Neisseria canis NCTC10296; Nbc, Neisseria baciliformis DSM 233383; Nmj, Neisseria mucosa ATCC 19696; Nani, Neisseria animaloris NCTC12227; Nsf, Neisseria subflava ATCC 49275; Nzo, Neisseria zoodegmatis NCTC12230; Nsg, Neisseria shayeganii DSM22244; Nsc, Neisseria sicca NS20201025; Nwd, Neisseria wadsworthii DSM22245; Nwe, Neisseria weaver NCTC13585. (PDF) [file ppat.1010497.s007.pdf]

S2 Table: A selection of genes identified as candidate host interaction factors

RefSeq Locus	GenBank Locus	Name	Annotation	Avg Fold Change	Pathway Association	*Neisseria* homologs⁺ (% Identity)	*Neisseria* gene ID
H7A79_RS9625	H7A79_1831	*A1/csiA*	UDP-N-acetylglucosamine epimerase	-2.2	capsule synthesis	Nzo(78.6), Nm*[1](72)	SAMEA4504057_2342, NMEN93004_0110 (86% q.c)
H7A79_RS09645	H7A79_1835	*A5*	hypothetical protein, putative rhamnosyl transferase	-3.0	capsule synthesis	Nci(64.8)	NCTC10296_00788
H7A79_RS09655	H7A79_1837	*A7)*	Glycosyltransferase	-4.4	capsule synthesis	Nm*[1](84), Nci(79)	**NMEN93004_0106** NCTC10296_00785
H7A79_RS03350	H7A79_0628	*mreD*	Rod shape-determining protein	-1.6	Type II TA system related factors	Nel(85.3), Nani(97.7)	NELON_06220, NCTC12227_00862
H7A79_RS03345	H7A79_0627	*mreC*	Rod shape-determining protein	-2.7	Chromosome partitioning proteins	Nel(62.0), Nsf(74.4), Nfv(55.1)	NELON_06225, FAH66_05595, FAH67_05500
H7A79_RS07140	H7A79_1361	*tig*	Trigger factor	-6.3	protein processing	Nzo(87.4), Nla(79.4), Nsc(79.6), Nmj(79.4), Ncz(79.1), Nme(79.6), Ngo(78.4)	SAMEA4504057_0773, NLA_11470, J7445_02895, NM96_05870, NCTC10294_00825, **NMB1313, NGO_0592**
H7A79_RS10380	H7A79_1976	*oxyR*	Hydrogen peroxide-inducible genes activator	-4.2	Transcriptional regulation	Nme(88.5), Ngo(88.2),Nel(84.6), Nsi(88.5), Nmj(88.9),Nsf(89.8), Nfv(89.5), Nla(88.8), Nani(88.7), Ncz(87.5)	**NMB0173**, **NGO_1813**, NELON_11430, A6J88_02495, NM96_10750, A6J88_02495, FAH67_10175, NLA_1150, NCTC12227_01942, NCTC10294_01691
H7A79_RS03110	H7A79_0582		tetR family transcriptional regulator	-3.1	Transcriptional regulation	Nzo(84.1), Nsf(79.4), Ngo(78.6), Nfv(78), Nla(77.7), Nme(76.8), Nsi(79.4)	SAMEA4504057_1144, FAH66_03145, **NGO_0393,** FAH67_03065, NLA14200, **NMB0810**, A6J88_12645
H7A79_RS07670	H7A79_1461	*bolA*	bolA family transcriptional regulator	-3.8	Transcriptional regulation	Nzo(77), Nmj(69.7), Nsi(69.7), Nfv(67.0), Nsf(65.9), Nel(60.2), Nla(56.2), Nme(59.5), Ngo(58.3)	SAMEA4504057_2161, NM96_10660, A6J88_02420, FAH67_06885, FAH66_00750, NELON_09195,

							NLA4050, **NMB0344, NGO_1657**
H7A79_RS14490	H7A79_2775		MarR family transcriptional regulator	-7.8	Transcriptional regulation	Nmj(81.5), Nsi(81.5), Nfv(78.5), Nel(53.1) Nsc(81.5)	NM96_13065, A6J88_04850, FAH67_00625, NELON_09340, J7445_11615
H7A79_RS01540	H7A79_0286	*matE*	Multidrug transporter	-3.2	Transport	Nsi(54), Nmj(53.3),Nsf(43.8), Nci(47.4), Nani(61)	A6J88_13235, NM96_07625, FAH66_00690, NCTC10296_00059, NCTC12227_01330
H7A79_RS03010	H7A79_0563	*gluP*	Glucose/galactose MFS transporter	-4.7	Transport	Nme(74.9), Ngo(74.4), Nsf(74.4)	NMB0535, **NGO_0142,** FAH66_06500
H7A79_RS04300	H7A79_0825	*mscL*	Large conductance mechanosensitive channel protein	-6.7	Transport	Nel(82.9), Npf(87.2), Nci(81.4), Ncz(80.1), Nmj(84.5), Nfv(84.4)	LPB400_02510, NCTC10296_01431, NCTC10294_00050, NM96_10410, FAH67_09185
H7A79_RS06400	H7A79_1225		Entericidin A/B family lipoprotein	-4.3	Transport	Nmj(65), Nani(88.6), Nci(81.8)	NM96_05900, NCTC12227_00917, NCTC10296_01715
H7A79_RS12155	H7A79_2323		oligopeptide ABC transporter substrate-binding protein OppA	-2.9	Transport	Nani(78.3), Nwe(78.2), Nzo(78.0), Nzl(76.8)	NCTC12227_01826, SAMEA3174300_2044, SAMEA4504057_0413, D0T92_02200
H7A79_RS14545	H7A79_2785		putative membrane protein/divalent metal cation transporter	-2.3	Transport	Nzo(85.7), Nsg(81.6), Nwd(88.3), Ncz(61.4), Nsf(61.1)	SAMEA4504057_0458, H3L94_00340, H3L96_03520, NCTC10294_01218, FAH66_01695
H7A79_RS05505	H7A79_1050	*cstA*	carbon starvation protein A	-4.8	Transport	Nzo(92.2), Nsf(85.3), Nel(85.7), Nbc(85.4), Nla(85.8), Nmj(85.2), Ncz(85.4), Nme(85.7)	SAMEA4504057_1672, FAH66_06180, NELON_08070, H3L91_03325, NLA_7830, NM96_01155, NCTC10294_00974, **NMB1493**
H7A79_RS00105	H7A79_0023	*cheY*	Response regulator	-3.2	Two component systems, motility	Nci(74.4), Nbc(74.1)	NCTC10296_01930, H3L91_02950
H7A79_RS00110	H7A79_0024	*cheX*	Chemotaxis protein CheX	-1.7	Two component systems, motility	Nani(83.1), Nci(72.5), Nbc(66)	NCTC1227_00276, NCTC10296_01931, H3L91_02945
H7A79_RS14225	H7A79_2721		response regulator	-8.1	Signal transduction	Nzo(82.1), Nsf(75.7),Nfv(76.0),	SAMEA4504057_2040, FAH66_00465,

						Nmj(73.2), Nsi(73.2), Nme(59.0)	FAH67_07230, NM96_13185, A6J88_04965, **NMB0476**
H7A79_RS10685	H7A79_2032		cyclic nucleotide-binding domain-containing protein	-2.2	signaling	Nwd(67.2), Nbl(60.1), Nzo(83.2)	H3L96_03595, GJV52_01670, SAMEA4504057_2064
H7A79_RS01525	H7A79_0283		vanZ family protein	-2.5	unknown	Ngo(55. 8), Nsi(56.0), Nsf(58.5), Nfv(57.6), Nmj(56), Nani(66.1)	**NGO_0378**, A6J88_11055, FAH66_04825, FAH67_04740, NM96_05350, NCTC12227_00265
H7A79_RS02820	H7A79_0527		yecA family protein	-7.0	unknown	Nzo(80.6), Nsf(71.5), Nfv(71.5), Nsi(71.6), Nla(66.4), Ngo(65.9), Nme(65.9)	SAMEA4504057_1302, FAH66_04555, FAH67_04490, A6J88_09255, NLA_9560, **NGO_0847, NMB1071**
H7A79_RS07070	H7A79_1349		Hypothetical/ conjugal transfer protein	-2.5	unknown	Nzo(80.1), Nel(61.8), Nsc(61.1), Nsf(58.2), Nfv(58.2), Ncz(57.8), Nla(58.2), Nme(57.9)	SAMEA4504057_01510, NELON_07995, J7445_06290, FAH66_05180, NCTC10294_00651, NLA_11020, **NMB1269**
H7A79_RS12320	H7A79_2356	*rhuM*	Virulence factor	-2.1 oral only	unknown	Nme(43.4)[1], Nfv(44)[1], Nel(44)[1], Nla(47.8)[1], Nmj(45.1)[1]	**NMB0830**(51% q.c), FAH67_09335(50% q.c), NELON_01635(14% q.c.), NLA_10660(43% q.c.), NMA96_02505(48% q.c.)
H7A79_RS13690	H7A79_2619		M61 family metallopeptidase	-1.6 oral & wk6	unknown	Ngo(65.1), Nme(64.7), Nmj(67), Nel(66.2), Nfv(64.8), Nla(64.4), Nsf(64.1), Nani(70.6), Nci(64)	**NGO_1375, NMB1726**, NM96_00550, NELON_10710, FAH67_07400, NLA_5450, FAH66_00370, NCTC12227_01620, NCTC10296_00269
H7A79_RS14210	H7A79_2718		roadblock/LC7 domain containing protein	-7.4	unknown	Nzo(96.7), Nmj(91.5), Nsi(91.5), Nfv(89.8), Nsf(88.1), Nla(78.0), Ngo(76.3), Nme(76.3)	SAMEA4504057_2037, NM96_13200, A6J88_04980, FAH67_07215, FAH66_00480, NLA_5120, **NGO_1475, NMB0479**

H7A79_RS04855	H7A79_0925	Rid family endoribonuclease	-3.7	Translational regulation	Nzo(91.3), Nel(84.9), Nmj(87.3), Nsc(87.3), Nsf(87.3), Ncz(87.3), Nsi(86.5), Nme(86.5), Ngo(83.3)	SAMEA4504057_1660, NELON_08435, NM96_07100, J7445_01830, FAH66_02245, NCTC10294_00295, A6J88_12725, **NMB0861, NGO_0232**

[+]Genes are grouped by putative pathway association identified by KEGG functional annotation. Amino acid sequence comparisons were computed from KEGG Ortholog Gene Function Identification (GFIT) Sequence similarity database tables using the Nmus Genbank ID. [1]Sequence analyzed by BLASTP to identify any similarity with human adapted *Neisseria*. Query coverage is reported for BLASTP comparisons. Species abbreviations are shown according to KEGG nomenclature. Nme, *Neisseria meningitidis* MC58; Nm*, *Neisseria meningitidis* serogroup I/93004; Nel, *Neisseria elongata sups. glycolitica* ATCC29315; Ngo, *Neisseria gonorrhoeae* FA1090; Nla, *Neisseria lactamica* 02-06; Nsi, *Neisseria mucosa* FDAARGOS; Nfv, *Neisseria flavescens* ATCC 13120; Ncz, *Neisseria cinerea* NCTC10294; Nci, *Neisseria canis* NCTC10296; Nbc, *Neisseria baciliformis* DSM 233383; Nmj, *Neisseria mucosa* ATCC 19696; Nani, *Neisseria animaloris* NCTC12227; Nsf, *Neisseria subflava* ATCC 49275; Nzo, *Neisseria zoodegmatis* NCTC12230; Nsg, *Neisseria shayeganii* DSM22244; Nsc, *Neisseria sicca* NS20201025; Nwd, *Neisseria wadsworthii* DSM22245; Nwe, *Neisseria weaveri* NCTC13585.
