# Supplementary material for: Neisseria genes required for persistence identified via in vivo screening of a transposon mutant library
Source: PLoS Pathog. 2022 May 17;18(5):e1010497. doi: 10.1371/journal.ppat.1010497 (PMC9140248; doi:10.1371/journal.ppat.1010497)
Supplement: S3 Table — Comparisons between library replicates 1 and 2 in each sample type, and between sample types in each replicate, were performed by Pearson r correlation analysis in GraphPad Prism V9. (PDF) [file ppat.1010497.s008.pdf]

S3 Table. Correlation of read counts and insertion indexes by replicate.

Comparison	Pearson r Insertion Index	r Confidence Interval Insertion index	Pearson r Read Count	r Confidence Interval Read Count
Inoculum rep. 1 v. rep. 2	0.988	0.9870 to 0.9887	0.996	0.9962 to 0.9967
Oral rep. 1 v. rep 2	0.962	0.9587 to 0.9642	0.954	0.9504 to 0.9569
Gut Week 6 rep. 1 v. rep. 2	0.980	0.9786 to 0.9814	0.938	0.9338 to 0.9425
Gut Week 8 rep. 1 v. rep. 2	0.948	0.9447 to 0.9520	0.934	0.9289 to 0.932
Oral v. Week 6 Gut rep. 1	0.986	0.9854 to 0.9874	0.938	0.9339 to 0.9426
Oral v. Week 8 Gut rep. 1	0.985	0.9840 to 0.9862	0.934	0.9294 to 0.9386
Oral v. Week 6 Gut rep. 2	0.981	0.9797 to 0.9824	0.958	0.9550 to 0.9609
Oral v. Week 8 Gut rep. 2	0.923	0.9176 to 0.9284	0.969	0.9665 to 0.9709
Inoculum v. Oral rep. 1	0.918	0.9122 to 0.9236	0.953	0.9491 to 0.9559
Inoculum v. Oral rep. 2	0.902	0.8951 to 0.9086	0.955	0.9520 to 0.9584
Inoculum v. Week 6 Gut rep. 1	0.929	0.9235 to 0.9335	0.971	0.9691 to 0.9732
Inoculum v. Week 6 Gut rep. 2	0.907	0.9009 to 0.9137	0.964	0.9611 to 0.9663
Inoculum v. Week 8 Gut rep. 1	0.922	0.9164 to 0.9273	0.939	0.9348 to 0.9433
Inoculum v. Week 8 Gut rep. 2	0.868	0.8588 to 0.8767	0.966	0.9636 to 0.9685
